# DNA methylation-regulated LINC02587 inhibits ferroptosis and promotes the progression of glioma cells through the CoQ-FSP1 pathway

**DOI:** 10.1186/s12885-023-11502-0

**Published:** 2023-10-17

**Authors:** Zhengang Wang, Yang Cui, Fanchen Wang, Lixia Xu, Yan Yan, Xiaoguang Tong, Hua Yan

**Affiliations:** 1 Clinical College of Neurology, Neurosurgery and Neurorehabilitation, Tianjin Medical University, Tianjin, 300350 China; 2 Department of Neurosurgery, Affiliated Hospital of Weifang Medical University, Weifang, Shan Dong 261000 China; 3 Department of Neurosurgery, Hebei Yanda Hospital, Langfang, He Bei China; 4 Cancer Molecular Diagnostics Core, Key Laboratory of Cancer Prevention and Therapy, Tianjin Medical University Cancer Institute and Hospital, National Clinical Research Center for Cancer, Tianjin’s Clinical Research Center for Cancer, Tianjin, 300060 China; 5 Tianjin Key Laboratory of Cerebral Vascular and Neurodegenerative Diseases, Tianjin Neurosurgical Institute, Tianjin Huanhu Hospital, Tianjin, 300350 China; 6 Clinical Laboratory, Tianjin Huanhu Hospital, Tianjin, 300350 China; 7 Department of Neurosurgery, Tianjin Huanhu Hospital, Tianjin, 300350 China

**Keywords:** Glioma, LncRNA, LINC02587, Methylation, Ferroptosis, RNA-seq, CoQ-FSP1

## Abstract

**Background:**

Long noncoding RNAs (lncRNAs) are considered key players in the formation and development of tumors. Herein, Gene Expression Profiling Interactive Analysis (GEPIA) was employed as a bioinformatics technology. LINC02587 is differentially expressed in bladder urothelial cancer, glioblastoma, lung adenocarcinoma, lung SCC, melanoma, and other tumor tissue and cells. However, its impact on the emergence of glioma and its mechanism is remaining elusive.

**Methods:**

Some of the in vitro assays employed in this study were the CCK-8 / Annexin-V / Transwell assays, colony formation, and wound healing, together with Western blot (WB) evaluation. MSP / BSP assays were employed for assessing the CpG island’s methylation status in the LINC02587 promoter. Through transcriptome, ferroptosis-related experiments, and WB evaluation, it was confirmed that LINC02587 is correlated with the regulation of ferroptosis in tumor cells, and CoQ-Fsp1 is one of its regulatory pathways. Moreover, the underlined in-vitro results were further validated by in-vivo studies.

**Results:**

The current study shows that the promoter sequence of LINC02587 is regulated by methylation. The silencing of LINC02587 can inhibit cellular proliferative, migrative, and invasive properties, and induce ferroptosis within gliomas through the CoQ-FSP1 pathway.

**Conclusions:**

LINC02587 is likely to be a novel drug target in treating glioma.

**Supplementary Information:**

The online version contains supplementary material available at 10.1186/s12885-023-11502-0.

## Introduction

Glioma is considered to be one of the most frequent tumors that develop in the nervous system. It makes up 24.7% of all primary intracranial tumors and 74.6% of all malignant tumors. It is responsible for a large number of neoplastic-related deaths worldwide. [1]. According to one recent study, brain tumors represent around 2% of all human cancers, with gliomas accounting for 60% of brain cancers [2]. Despite advancements in treatment, the survival rate for those with gliomas is still low; especially the 5-year survival rate of glioblastoma, which is 0.05–4.7%, and the median survival rate is 14.6–17 months [3]. To uncover effective therapy strategies, it will be necessary to investigate the underlying molecular pathways implicated in glioma proliferative properties.

Genomes are extensively transcribed by RNA polymerase II, resulting in the production of thousands of long non-coding RNAs (lncRNAs), which are defined as RNAs containing between 200 and 100,000 nucleotides and lacking an effective open reading frame [4]. Although lncRNAs cannot encode proteins, their function directly with RNAs, and their production process are similar to that of protein-coding genes. lncRNAs play a key role in the occurrence and progression of numerous malignant tumors [5]. Multiple studies on malignant tumors have shown, in particular, that certain lncRNAs have a discernible role in mRNA transcription or translation by reducing, silencing, or overexpressing mRNA transcripts, thus affecting the proliferation, differentiation, apoptosis, and invasion of malignant tumor cells [6, 7].

Both disruption in gene expression and the emergence of many human malignancies are significantly influenced by epigenetic biomarkers. Numerous studies have demonstrated that gene expression silencing may be directly induced by hypermethylation of gene promoter regions [8]. Multiple studies have recently reported that CpG island hypermethylation within the promoter region can affect anti-sense lncRNAs together with sense-based genes [9–13].

**Fig. 1:**
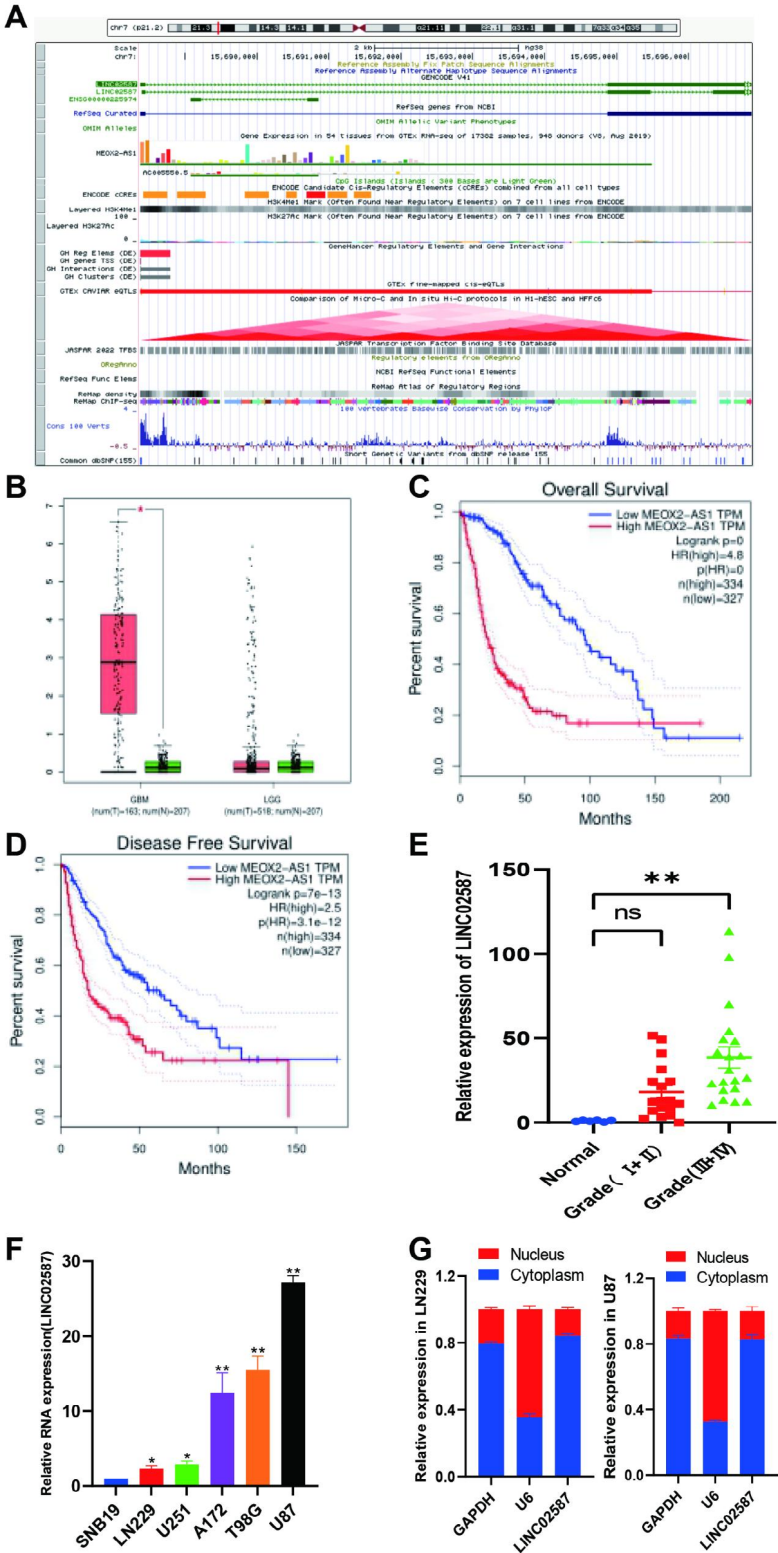
The expression of LINC02587 in glioma. (**A**) Genomic characterization of LINC02587, cited from UCSC.(**B**) GEPIA analysis of LINC02587 expression in GBM and LGG patients compared with that in normal brain tissues.(**C**) GEPIA analysis of the prognostic significance of LINC02587 expression and OS in glioma patients.(**D**) The relationship between LINC02587 expression and DFS in glioma patients.(**E**) Relative expression level of LINC02587 in glioma malignant tissues and paired normal tissues. Normal(6 cases), Grade I-IV (grade I+II 18 cases, III+ IV 20 cases). (**F**) Relative expression level of LINC02587 in 6 glioma cell lines. (**G**) The levels of LINC02587 in nuclear and cytoplasmic fractions of LN229 and U87 cells detected by qRT-PCR. GAPDH cytoplasmic control; U6 nuclear control. Data are expressed as the mean ± SD.*P < 0.05.**P < 0.01

The analyses of GEPIA databases revealed that LINC02587 is significantly correlated with a variety of tumor sites;LINC02587 is located on 7p21.2 and contains some CpG islands(Fig. 1A). However, no relevant information regarding its association with glioma was obtained. According to the reported study by Liu et al., MEOX2-AS1 (also called LINC02587) levels were markedly upregulated in cervical cancer cells and tissues, and higher MEOX2-AS1 expressions indicated a poor clinical outcome [14]. In contrast, MEOX2-AS1 silencing suppressed the metastatic and proliferative ability of cervical cancer cells. Ouyang et al. reported that MEOX2-AS1 expression in residual cancer tissues was significantly lower than that in remittent tissues [15].

This study aimed to investigate the correlation between LINC02587 expression and the clinicopathological characteristics of glioma patients, as well as their underlying mechanisms.

## Materials and methods

### Human samples

Overall, 6 cases of non-tumor brain tissue, 18 cases of grade I-II glioma samples, and 20 cases of grade III-IV glioma samples were obtained from Tianjin Huanhu Hospital, China. Non-tumor brain tissue was obtained from cases that needed post-trauma surgery due to traumatic cerebral injury or cerebral hemorrhage. While glioma tissue was procured through the neurosurgical institute sample library. The Tianjin Huanhu Hospital Ethics Committee approved this study. Each participant was aware of the study’s objectives and provided a signed/written informed consent form.

### GEPIA database

GEPIA (http://gepia.cancer-pku.cn/index.html) [16] was employed to interpret and assess LINC02587 expression profiling and its prognostic significance within glioma cases and non-tumor-control individuals. To validate LINC02587 expression and survival evaluation, LGG (low grade glioma; n = 518), GBM (glioblastoma multiform; n = 163), and non-tumor-control (n = 207) cohorts were employed.

### Cell culture and transfection

Peking Union Medical College (Beijing, China) supplied U87, T98G, and LN229 cells, while iCell Bioscience Inc. Co. Ltd. supplied SNB19, A172, and U251 (Shanghai, China). These cell lines were incubated at 37 °C in a humidified incubator with a constant supply of 5% CO_2_ MEM or DMEM (Thermo Fisher Scientific) containing 10% FBS (Thermo Fisher Scientific). GenePharma (Shanghai, China) provides both specific and non-specific siRNA (si-NC) for transfection of siRNAs targeting LINC02587 (si-LINC02587-i, si-LINC02587-ii, and si-LINC02587-iii). The siRNA sequences are as follows: si-NC, 5′-UUCUCCGAACGUGUCACGUTT-3′; si-LINC02587-i, 5′-GCAAGUCUGACGGUACCAATT-3′; si-LINC02587-ii, 5′-GGGUCACAUGCCUGGUGUUTT-3′; si-LINC02587-iii, 5′-GCUCAUGGACCAGAACAAATT-3′. As per the kit protocol, Lipofectamine 2000® (Invitrogen™, Carlsbad, USA) was employed to transfect the LN229 and U87 cells.

### Quantitative real-time PCR (RT-qPCR)

TRIzol reagent (Thermo Fisher Scientific) was used to isolate total RNA from human glioma tissues and glioma cells, following the instructions from the manufacturer. Next, 1 µg of RNA was reverse transcribed using the SureFireRT TM kit (06-104; abgen, Tianjin, China). qRT-PCR was performed using GreenHOToGo TM Taq Mix (05-110121; abgen). The details for primer sequences were shown in supplementary file 1: Table S1. The PCR reaction was performed by an initial incubation at 95 °C for 5 min, followed by 50 cycles of 95 °C for 20 s, 60 °C for 20 s, and 72 °C for 20 s using a Roche Lightcycler 480II (Roche, Basel, Switzerland). Furthermore, the relative gene expressions were evaluated by the 2−∆∆CT method and normalized to that of GAPDH.

### Cell viability assay

LN229 and U87 cells were grown within 96-well plates (5000/well) in triplicate and incubated for 24-, 48-, and 72-h post-transfection using 100 µL/L of si-NC or si-LINC02587. Then, each well received 10 µL of the CCK-8 (K009-500®; ZETA Life™, Menlo Park, USA), which was then placed into incubation (1–4 h; 37 °C). Finally, the O.D. was recorded (450 nm), through a microplate reader (Molecular Devices™, San Jose, USA).

### Cell apoptosis assay

LN229 and U87 cells transfected with si-NC or si-LINC02587 were harvested and suspended in 100 µL of binding buffer (0.1 M HEPES, pH 7.4, 1.4 M NaCl, and 25 mM CaCl_2_) at a density of 1 × 10^6^ cells/mL. The cell suspension was then mixed with Annexin V FITC Conjugate and propidium iodide (PI) solution and incubated for 15 min at room temperature in the dark. The apoptotic cells were detected and analyzed using a BD Caliber flow cytometer (BD Biosciences). The total percentage of Annexin V-positive cells was calculated quantitatively.

### Colony formation assay

To evaluate the colony-forming activity, the si-NC or si-LINC02587-transfected U87 and LN229 cells were seeded into 6-well plates (500 cells per well) and grown for 10 days to form colonies. Next, the cells were stained with 0.1% crystal violet (Sigma-Aldrich, St Louis,

USA) and examined under a microscope (Nikon, Tokyo, Japan) for observation and image recording.

### Wound healing analysis

For the wound healing assay, cells were cultured in 12-well plates. Next, the bottoms of the wells were scratched with a pipette tip. After being washed twice, cells were cultured within the serum-free medium (SFM) for 24 h. Finally, the wound closure was measured in percentage (%) by using ImageJ.

### Invasion assays

For invasion assays, U87 and LN229 cells (5 × 10^4^ cells/well resuspended in 200 mL MEM or DMEM) were added to the 24-well upper chambers (8-µm pore size; Corning Co., Corning, USA) with Matrigel (BD Biosciences). Next, 600 µL of DMEM containing 10% FBS was added to the bottom chamber. After 24 h of incubation, the culture medium in the upper chamber was discarded and the non-migrated cells were removed. The cells passed through the membrane were fixed with methanol and stained with a 1% crystal violet staining solution (Sigma-Aldrich). Additionally, five randomly selected fields from various angles were used for cell counting, image capturing, and observation under a 400x magnification microscope.

### Subcellular fractionation

Through employing an RNA-isolating system (PARIS™Invitrogen), nuclear plasma separation was carried out in LN229 and U87 cells, as previously described [17]. For nuclear and cytoplasmic controls, U6 and GAPDH were utilized, accordingly.

### 5-Aza-dC treatment

Three cohorts of LN229 or U87 cells, blank (no medication), 5-Aza-dC (5 µM), and 5-Aza-dC (10 µM) treatment cohorts were made. It took 72 h to treat 5 × 10^5^/mL cells, and the medium containing the new medicine was changed every 24 h.

### BSP and methylation specific PCR (MSP) assay

Sangon Biotech performed the MSP and BSP assays and data evaluation (Shanghai, China). DNA was isolated from U87 and LN229 cells, followed by bisulfate modification. Then, the obtained DNA was employed for the MSP and BSP assays. BSP and MSP assays were employed to analyze and sequence the promoter regions 1 (from 357 to 690; including 20 CpG sites) and 2 (from 667 to 998; including 17 CpG sites), shown in Table S2 of Supplementary File 1 (S2).

### Western blot analysis

Using RIPA lysis buffer and PMSF (Solarbio, Beijing, China), proteins were extracted from si-NC- or si-LINC02587-transfected U87 and LN229 cells following the manufacturer’s instructions. Next, the proteins were separated by 10% SDS-PAGE and transferred to a nitrocellulose membrane (Thermo Fisher Scientific). And the blots were cut to a suitable size prior to binding with antibodies during blotting. The proteins were then blocked with 5% bovine serum albumin (BSA, Solarbio), followed by incubating with primary antibodies (1:1000) against Bcl-2, Bax, MMP2, N-cadherin, ZEB1, ZO1, FSP1, CoQ10B, or β-actin. All antibodies were purchased from Cell Signaling Technology (Danvers, USA) except FSP1**(**DF8636, Affinity Biosciences**)**and CoQ10B (bs-11656R, Bioss). After a thorough wash, the membrane was incubated with horseradish peroxidase-conjugated goat anti-rabbit/mouse IgG secondary antibody (1:5000; Zhongshan Jinqiao Biotechnology Co., Ltd, Beijing, China) at room temperature for 90 min. Then enhanced chemiluminescence kit was used to visualize the blots on the membrane. Finally, the blots were quantified by densitometric analysis using ImageJ software. In this study, **β**-actin was used as the loading control.

### Immunohistochemistry (IHC)

The paraffin-embedded brain tissues were sliced, and IHC was performed using a YN-05MY automatic immunohistochemical staining system (Shenzhen Yongnian Technology) with primary antibodies Ki67 (1:500, ab16667, Abcam, UK) and FSP1(1:500, DF863, Affinity Biosciences). Immunostained sections were imaged with a positive fluorescence microscope (Carl Zeiss). The integrated optical density (IOD) value of the IHC section as well as the size and density of the stained region were measured using Image-Pro Plus version 6.0 software. The mean densitometry of the digital image (with 400x magnification) was designated as representative Ki67 or FSP1 staining intensity (indicating the relative Ki67 or FSP1 expression level). Five fields were randomly selected, and the tissue area signal densities were counted and statistically analyzed.

### Evaluation of mitochondrial membrane potential (MMP)

Using an MMP assay for JC-1 (Beyotime™, Shanghai, China), the mitochondrial membrane potential was measured in line with kit protocol.

### Evaluation of lipid peroxidation

According to the manufacturer guidelines, BODIPY 581/591 C11 (GLPBIO™, CA, USA) was employed for quantifying the level of lipid peroxidation.

### Evaluation of cellular iron concentration

The manufacturer’s instructions were followed when using the iron assay kit (FerroOrange®, F374, Dojindo Molecular Technology™) for measuring the level of cellular iron.

### Glutathione assay

GSH Detection Kit (Solarbio Co., Beijing, China) was used to detect reduced glutathione (GSH) according to the manufacturer’s instructions.

### RNA sequencing

The Shanghai Applied Protein Technology™ Co., Ltd. has completed RNA sequencing (RNA-seq) and data evaluation (Shanghai, China). The Si-NC or si-LINC02587-ii transfected LN229 glioma cells were collected for their total RNA assessments.

### The Nude murines xenograft model

Male, 4-week-old BALB/c-A nude murines were procured through Sibeifu Beijing Biotechnology™ Co. Ltd. (China). The Tianjin Huanhu Hospital’s Animal Care and Use Committee gave its approval for the use of animals in assays. The two cohorts of nude murines (Scramble-shRNA and hLINC02587-shRNA; n = 5 in each cohort) were randomly assigned. The right limbs of the nude murines received subcutaneous injections of 5 × 10^6^ U87 cells that underwent stable transfection with either hLINC02587-shRNA or Scrambled shRNA and 100 µL of PBS. After tumor inoculation, the growth of the tumor was checked weekly for a total of five weeks using a caliper. The formula employed to determine tumor volume was V = 0.5 × length × breadth^2^. All nude murines were euthanized(They were first given sedative,1.5% pentobarbital sodium, 0.04ml/10 g, intraperitoneal injection, and then sacrificed by cervical dislocation)5 weeks later, and the tumor masses were taken out and weighed.

### Statistical evaluation

GraphPad Prism software was employed for the statistical evaluation.

The Student’s t-test was employed to make a comparison across both cohorts, whereas ANOVA was applied to compare ≥ 3 cohorts. P-values < 0.05 were deemed to confer statistical significance.

## Results

### LINC02587 upregulated within gliomas, and it was linked to a poor prognosis

GEPIA dataset outcomes revealed LINC02587 significant upregulation within GBM patients than in non-tumor brain tissue (Fig. 1B). Upregulated LINC02587 was linked to scant overall survival (OS; P = 0) and disease-free survival (DFS; P = 3.11012) in glioma patients, as determined by an evaluation of a glioma cohort utilizing GEPIA that included GBM (n = 334) and LGG (n = 325) (Fig. 1C-D). Overall, these dataset outcomes demonstrated that upregulated LINC02587 is linked to poor prognosis. LINC02587 expression was confirmed in glioma patients (Fig. 1E) and cell lines using RT-qPCR in this study (Fig. 1F). The two malignant glioma cell lines (LN229 and U87), which were among the six investigated cell lines (i.e., T98G, A172, U251, SNB19, LN229, and U87), displayed peak/trough levels for LINC02587 expression, accordingly (Fig. 1F). As a result, the biological role of LINC02587 in glioma cells was examined using U87 and LN229 cells. The RT-qPCR study revealed that LINC02587 was mostly expressed in the cytoplasm of U87 and LN229 cells, indicating important functions in the cytoplasm (Fig. 1G).

### Aberrant DNA methylation-regulation of LINC02587 in glioma

**Fig. 2:**
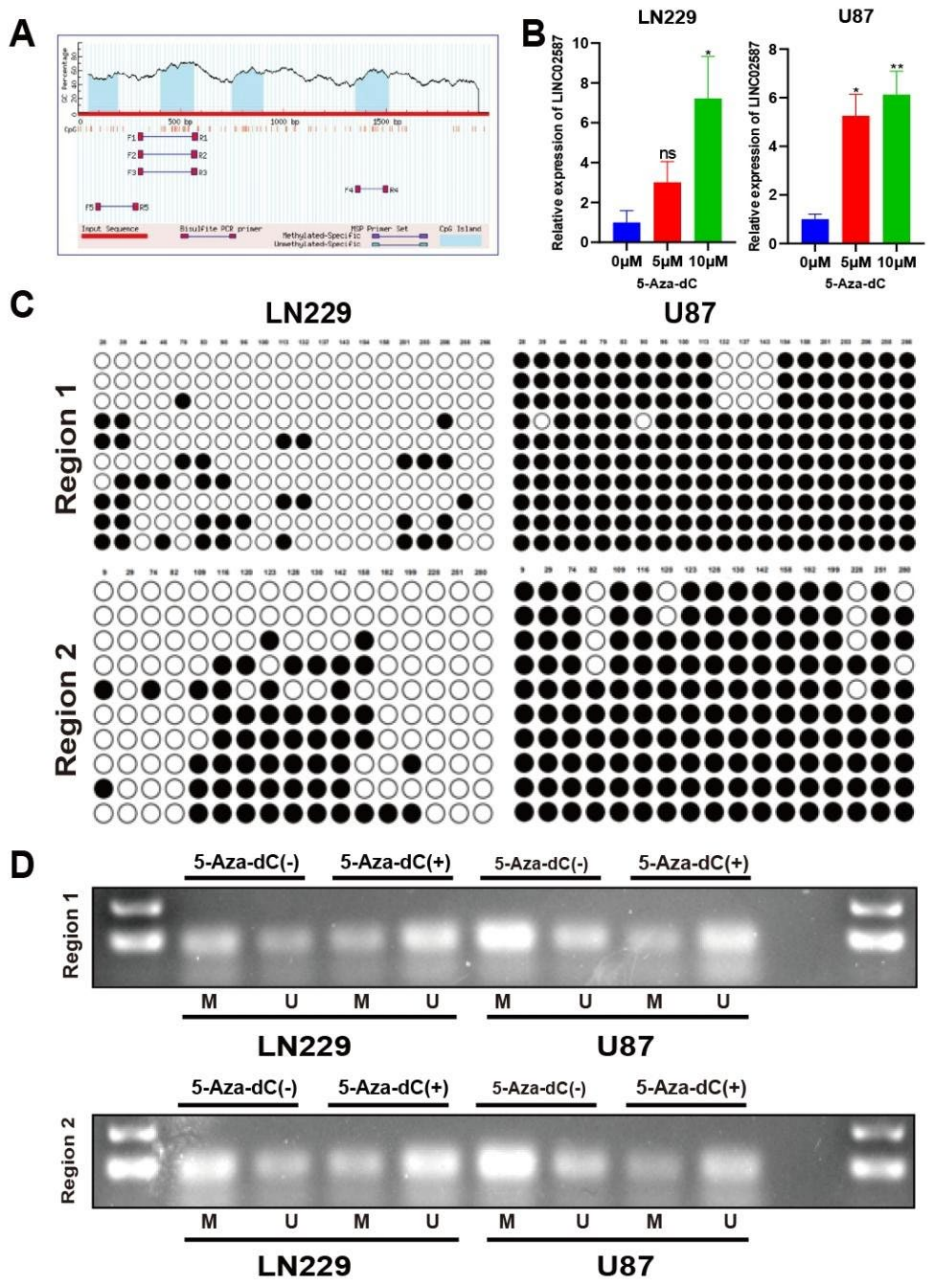
Assays to determine the epigenetic regulation mechanism of LINC02587 in glioma. (**A**) Schematic structure of CpG islands predicted by MethPrimer. The MSP regions analysed are indicated.(**B**) The expression level of LINC02587 in glioma cell lines treated with 5-Aza-dC measured by qRT-PCR.(**C**) Mapping of the methylation status of every CpG site of LINC02587 in 2 glioma cancer cell lines by BGS. Each CpG site was shown in the top row as an individual number. The different colours of circles for each CpG site represent the status of methylation(blank: unmethylation, black:methylation).(**D**) The methylation status of 2 regions of LINC02587 detected by MSP analysis in 2 cancer cell lines before and after 5-Aza-dC treatment(“M” represents methylated primers and “U” represents unmethylated primers).Data are expressed as the mean ± SD.*P < 0.05, **P < 0.01.ns, not significan

The current study performed a genomic region search using the UCSC Genome Browser to evaluate the underlying signaling pathway of LINC02587. Herein, the current study revealed 4 CpG islands in the promoter of LINC02587 (Fig. 2A). It was proposed that LINC02587 expression could be impacted by DNA methylation alteration. Additionally, the glioma cells were treated with DNA methyltransferase inhibitors, i.e., 5-Aza-dC. Figure 2B depicts LINC02587 expression profiles in 5Aza-dC cohorts increased along with the concentration of 5-Aza-dC. These findings showed that DNA methylation may control the expression of LINC02587. We examined the DNA sequence of the LINC02587 promoter CpG island and created primers for both MSP and BSP tests to confirm the methylation situation of the region. The promoter region 1 (357–690; including 20 CpG sites) and region 2 (667–998; including 17 CpG sites) methylation status was validated using the BSP assay (Fig. 2C). In U87 and LN229 cells, the methylation status was further assessed using the MSP approach both pre-/post-5-Aza-dC introduction. Before 5-Aza-dC treatment, region 1 and region 2 were found to be methylated; however, the aberrant methylation was partially restored post-5-Aza-dC introduction (Fig. 2D). These dataset outcomes suggested that one of the key pathways for LINC02587 activation and potential involvement in glioma progression may be hypermethylation of the promoter CpG island.

### Silencing LINC02587 reduces glioma cellular proliferative properties and drives apoptotic activity

**Fig. 3:**
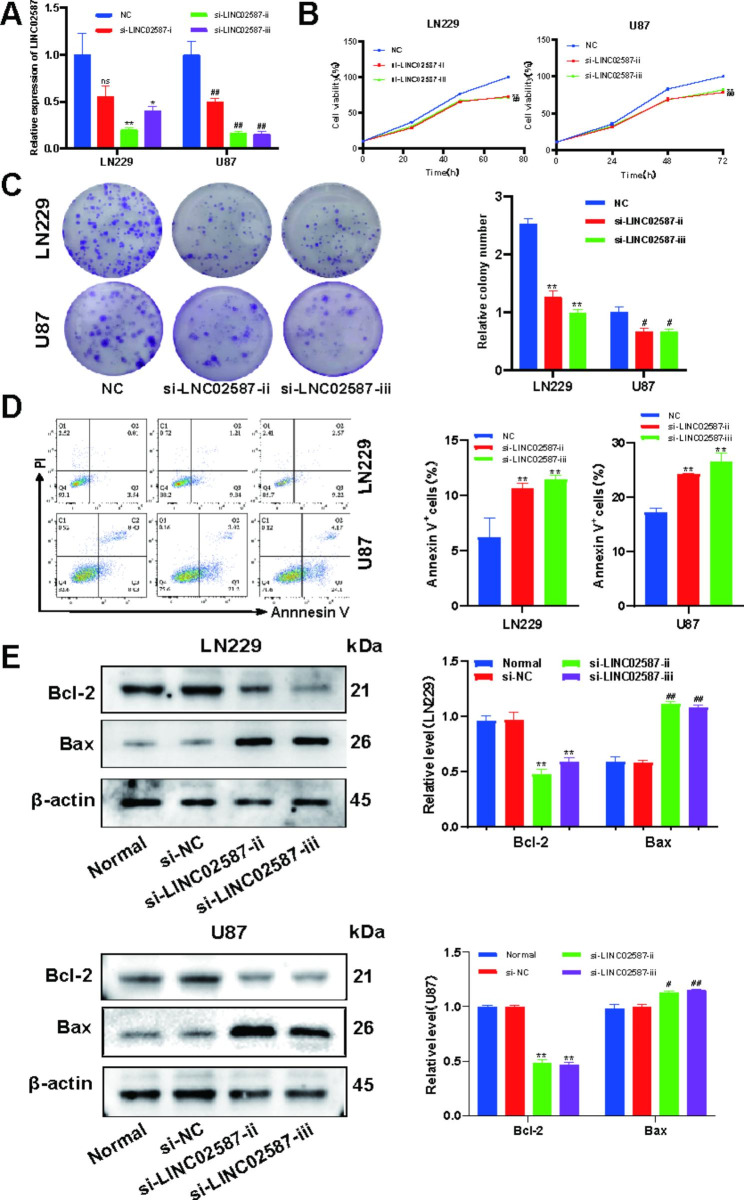
LINC02587 silent expression suppresses glioma cell roliferation and induces apoptosis. (**A**) si-LINC02587-i ,si-LINC02587-ii and si-LINC02587-iii caused obvious downregulation of LINC02587 in LN229 and U87 cells. (**B**) The number of viable cells(LN229 and U87) was determined by the CCK-8 assay after transfection with si-NC or si-LINC02587.(**C**) A colony formation assay was carried out to analyse the cell proliferation ability. Cells transfected with si-LINC02587 had decreased colony formation. (**D**) The affects of LINC02587 on LN229 and U87 cell apoptosis were determined by the Annexin V-FITC/PI assay. (**E**) Downregulation of LINC02587 expression increased the expression of Bax protein and suppressed the expressions of Bcl-2 in U87 and LN229 cells.Data are expressed as the mean ± SD. *P < 0.05, **P < 0.01, ^##^P < 0.01 vs. NC/si-NC group

To investigate the role of LINC02587 within glioma proliferation, U87 and LN229 cells were transfected with three siRNA molecules aimed against LINC02587, si-LINC02587-i, si-LINC02587-ii, and si-LINC02587-iii together with a si-NC control, for silencing LINC02587 gene. LINC02587 siRNA therapies reduced LINC02587 RNA levels, particularly si-LINC02587-ii and si-LINC02587-iii (Fig. 3A). The CCK-8 assay revealed that they had a modest growth rate (Fig. 3B). A colony formation assay revealed that LINC02587 downregulation thwarted U87 and LN229 (Fig. 3C). Cells transfected with si-LINC02587 had a considerably higher population of Annexin V-positive cells, depending upon an Annexin V-FITC/PI assay (Fig. 3D). We next employed western blot evaluation for further probing of proteomic dysregulation for genes involved in cell apoptotic activity. When glioma cells were transfected with si-LINC02587 as opposed to si-NC, Bcl-2 expression levels were dramatically decreased and Bax expression levels were significantly enhanced (Fig. 3E). Together, these findings demonstrated that LINC02587 silencing inhibits the proliferative of glioma cells and triggers apoptotic activity in those cells.

### LINC02587 silencing prevents migrative and invasive gliomas

**Fig. 4:**
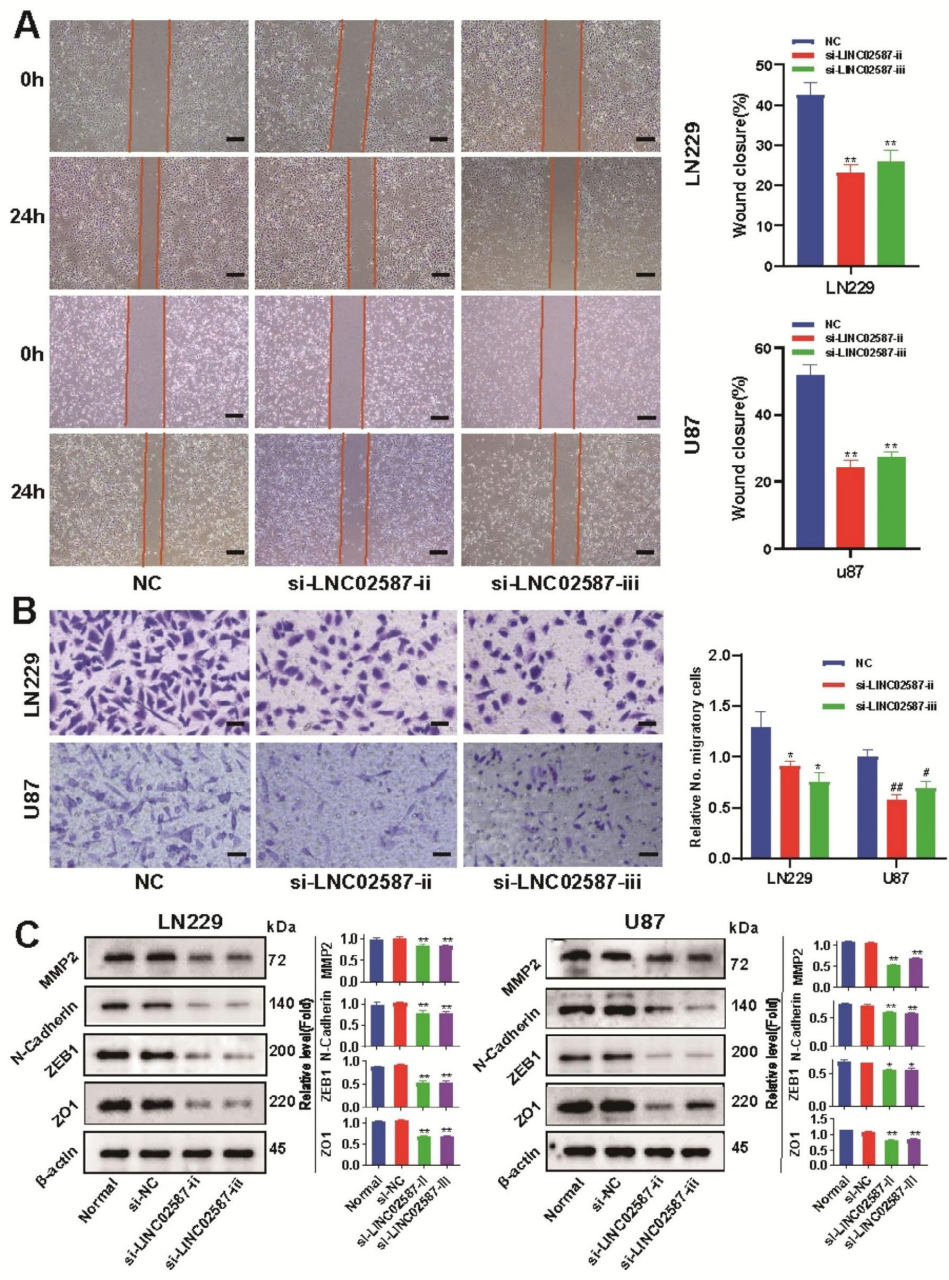
LINC02587 silencing inhibits glioma invasion and migration. (**A**) Migration ability of U87 and LN229 cells transfected with si-NC or si-LINC02587 was analyzed using wound-healing assay.Scale bar, 250 μm. (**B**) Invasive ability of U87 and LN229 cells transfected with si-NC or si-LINC02587 was evaluated by transwell assay. Scale bar, 100 μm. (**C**) Expressions of EMT markers in U87 and LN229 cells transfected with si-NC or si-LINC02587. Data are expressed as the mean ± SD. *P < 0.05,**P < 0.01, ^#^P < 0.05, ^# #^P < 0.01 vs. NC/si-NC group

Herein, the si-LINC02587-transfected cells were employed to evaluate the in-vitro migrative/invasive assays to reveal how LINC02587 knockdown affected the migrative and invasive U87 and LN229 cells (Fig. 4A, B). Cell migrative (Fig. 4A) and invasive (Fig. 4B) were reduced in si-LINC02587-transfected cells compared to si-NC-transfected cells, indicating that LINC02587 knockdown in vitro. Western blot evaluation was employed for observing the EMT process within LINC02587-depleted glioma cells. The mesenchymal markers levels of protein MMP2 and N-cadherin protein, ZO1 (tight junction marker), and ZEB1 (EMT-related transcription factor) levels were reduced in comparison to controls (Fig. 4C). Such findings suggest that silencing LINC02587 decreases glioma invasive/migrative properties.

### Downregulation of LINC02587 inhibits the CoQ-FSP1 pathway

**Fig. 5:**
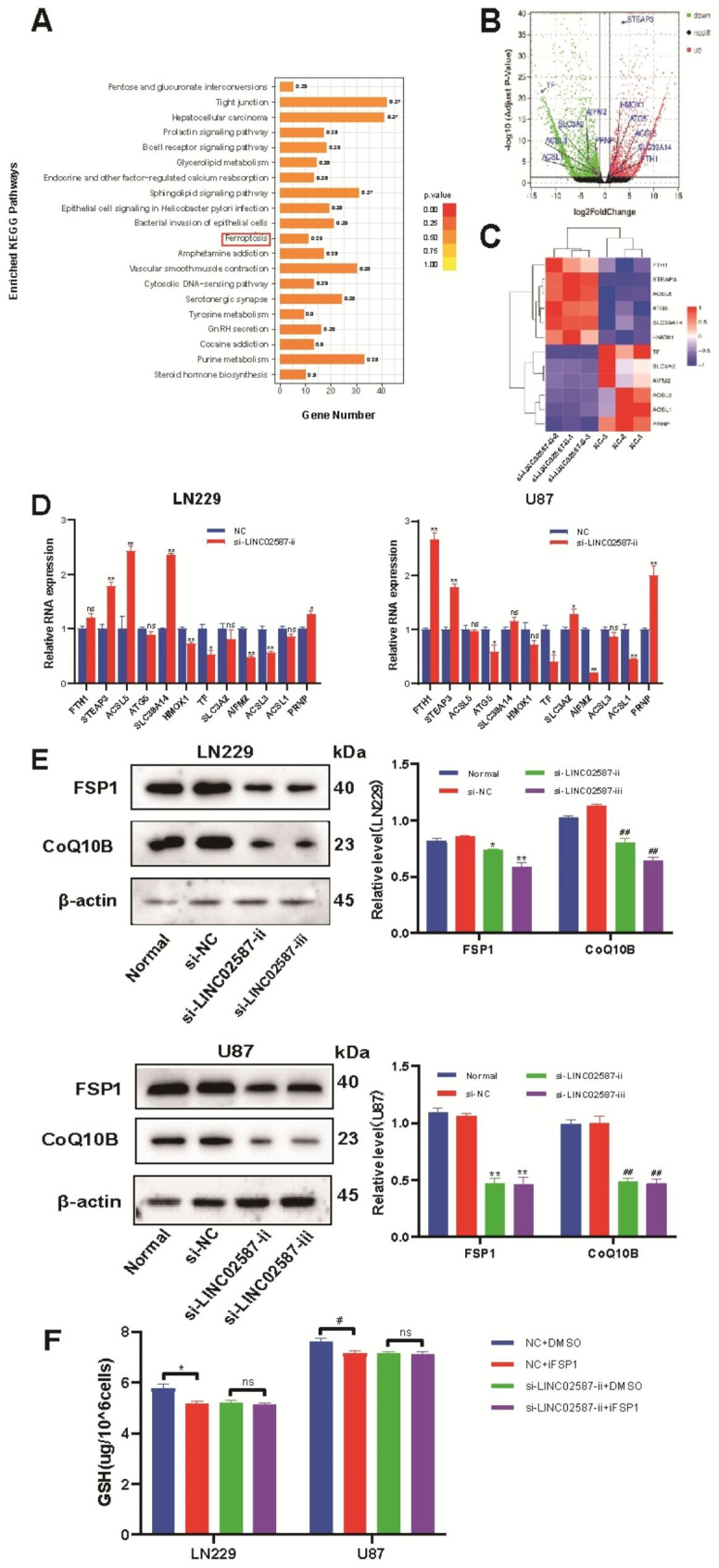
LINC02587 silencing inhibits the CoQ-FSP1 pathway. (**A**) KEGG pathway enrichment in the si-NC and si-LINC02587-ii groups. (**B**) Volcano map of differentially expressed Ferroptosis-related genes.(**C**) Hierarchical clustering heatmap of differentially expressed Ferroptosis-related genes.(**D**) qRT-PCR was carried out to analyse the relative expression of Ferroptosis-related genes in the si-NC and si-LINC02587-ii groups. (**E**) Western blot analysis of CoQ10B, FSP1 in U87 and LN229 cells transfected with si-NC or si-LINC02587. (**F**) Changes in GSH levels in cells after iFSP1 and si-LINC02587-ii intervention.Data are expressed as the mean ± SD.*P < 0.05,**P < 0.01, ^#^P < 0.05,^# #^P < 0.01 vs. NC/si-NC group

RNA-seq analyses were employed on LN229 cells transfected with si-NC or si-LINC02587-ii to examine the mechanism of LINC02587 in glioma (Fig. 5A–C). Ferroptosis was one of the signaling pathways identified by KEGG enrichment evaluation as being related to cancer (Fig. 5A). Volcano plot evaluation with a log 2 (fold-change) > 1 and P-value < 0.05 screening criterion revealed 12 ferroptosis-related genes that were differentially expressed (Fig. 5B). The samples were split into two main cohorts according to a heatmap produced by cluster evaluation (Fig. 5C). The LN229 or U87 cells were divided into two cohorts, si-NC, and si-LINC02587-ii cohorts. Moreover, RT-qPCR analyses were employed to evaluate the relative expression of the 12 ferroptosis-related genes(Fig. 5D). Among these, AIFM2 (also known as FSP1) has a clear difference in expression, which is consistent with the outcome predicted by transcriptomics. By using western blot evaluation, we were able to identify changes in FSP1 and COQ10B protein expression in glioma cells, which were crucial to the proliferative of these cancerous cells. The outcomes have shown that si-LINC02587 transfection massively reduced the levels of FSP1 and CoQ10B in both U87 and LN229 cells when compared to si-NC transfection (Fig. 5E). We selected the specific inhibitor of FSP1, iFSP1 (MCE, HY-136,057), to interfere with the CoQ-FSP1 pathway to confirm the mechanism of LINC02587,after the intervention of iFSP1(3 µ M; Incubation for 24 h), the level of Ferroptosis of tumor cells increased significantly in two tumor cell lines (LN229 and U87); After si-LINC02587-ii transfection and iFSP1(3 µ M; Incubation for 24 h) intervention, the level of Ferroptosis of tumor cells did not change significantly(Fig. 5F).These findings suggested that in glioma cells, LINC02587 knockdown suppresses the CoQ-FSP1 pathway.

### LINC02587 silencing increases oxidative stress induced by ferroptosis in glioma cells

**Fig. 6:**
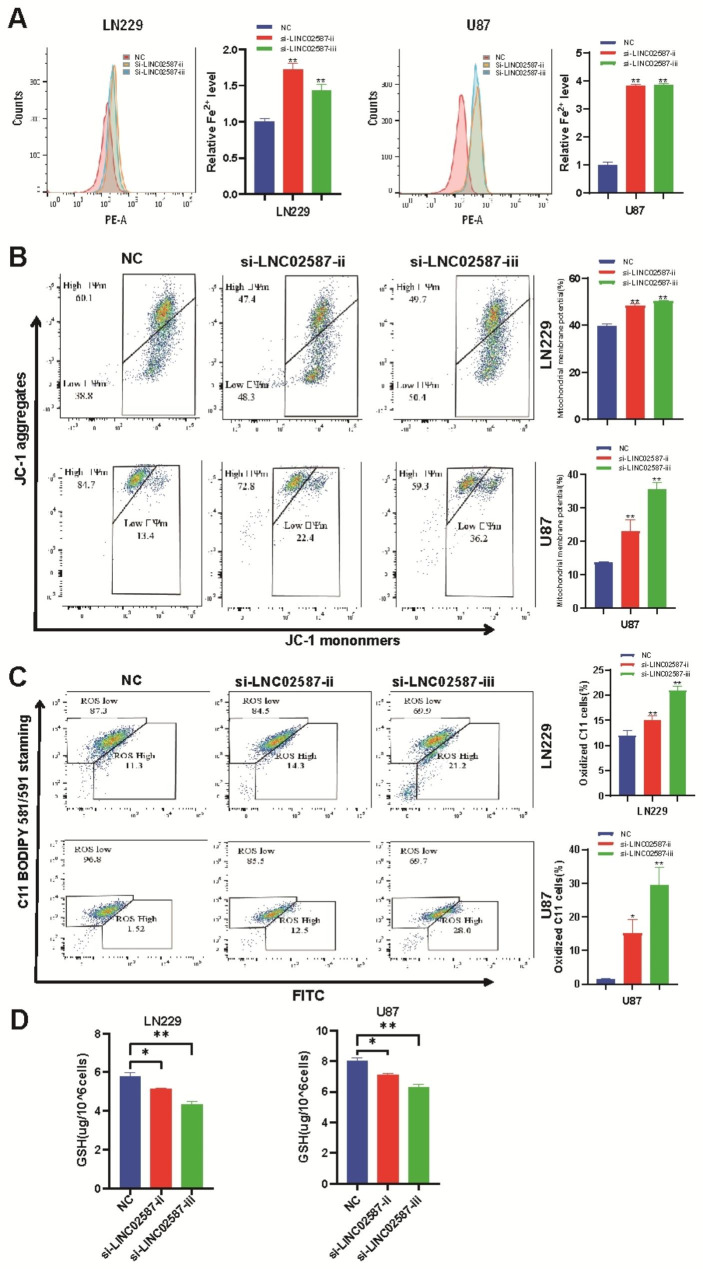
The association of LINC02587 and ferroptosis in glioma. (**A**) The Fe2 + concentration was measured by FerroOrange probe.(**B**) Mitochondrial membrane potential of U87 and LN229 cells transfected with si-NC or si- LINC02587.(**C**) Lipid peroxidation was detected in glioma cells, using C11 BODIPY 581/591 probe on flow cytometry. (**D**) The reduced GSH level of cells. Data are expressed as the mean ± SD.*P < 0.05,**P < 0.01 vs. NC group

Ferroptosis-related oxidative stress indicators including Fe^2+^ concentration, mitochondrial membrane potential, and lipid peroxidation were measured in glioma cells to validate the transcriptome evaluation dataset outcomes. FerroOrange regent detected the Fe^2+^ level. According to the obtained dataset outcomes, the si-NC cohort’s iron levels were reduced than those of the LINC02587 deficiency, which induced higher iron levels (Fig. 6A). Ferroptosis, which has a strong correlation to mitochondrial membrane potential and can be detected by the JC-1 sensor. The mitochondrial membrane potential in the LINC02587 knockdown cohort was found to be considerably reduced in comparison to the control cohort (Fig. 6B). The C11 BODIPY 581/591 probe detects lipid peroxidation, which is a critical indication of ferroptosis. The LINC02587 knockdown cohort’s lipid peroxidation considerably increased as compared to the control cohort (Fig. 6C) ; Compared with control group, GSH levels (Fig. 6D) were markedly reduced in the mimic group;Dataset outcomes showed that LINC02587 knockdown was found to enhance ferroptosis-related oxidative stress in glioma cells in vitro.

### Silencing of LINC02587 inhibits glioma tumorigenesis in vivo

**Fig. 7:**
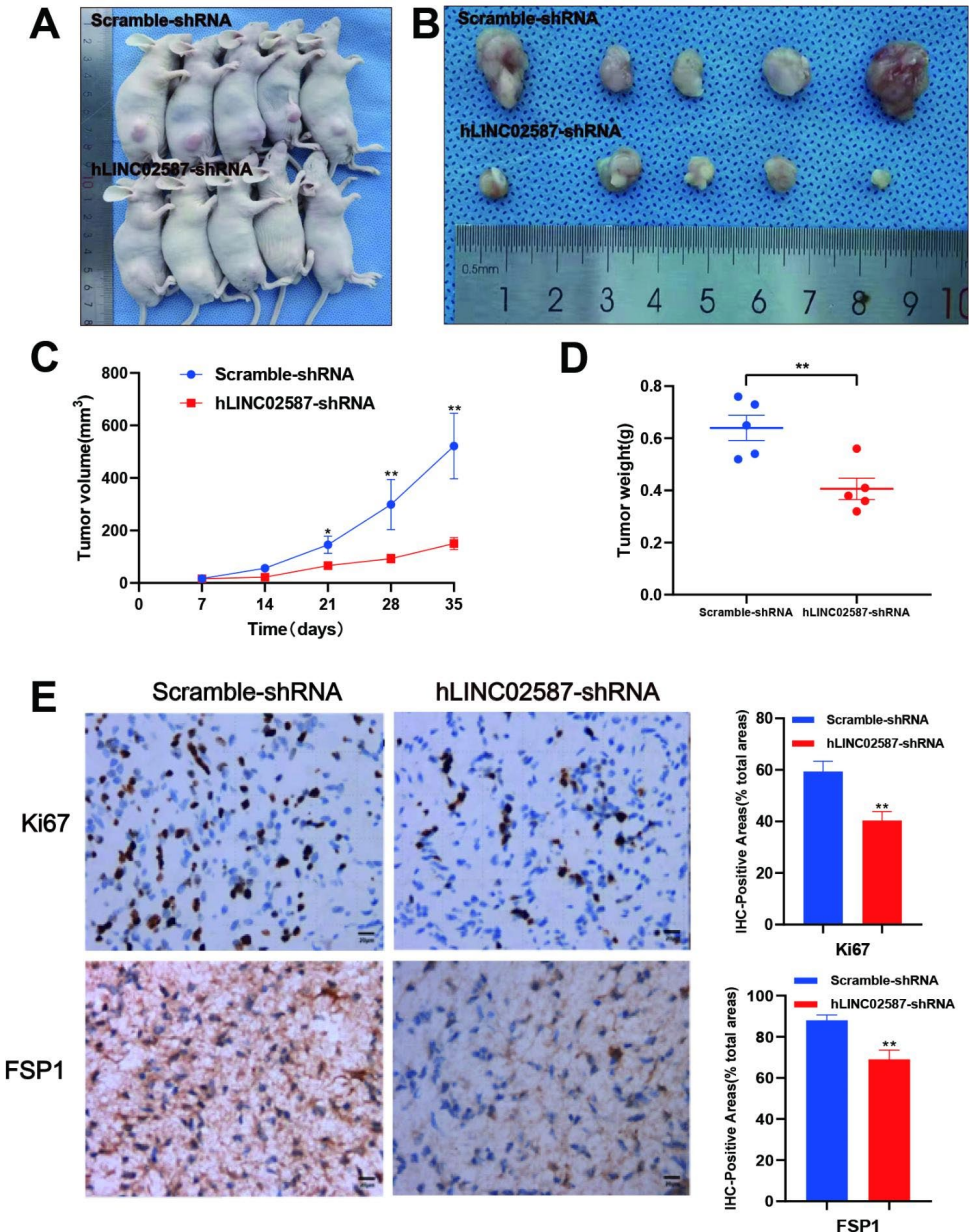
LINC02587 silencing inhibits glioma tumorgenesis in vivo. (**A**) External whole body images of nude mice killed 5 weeks after initial injection. (**B**) Representative images showing subcutaneous tumors of nude mice in the indicated two groups. (**C**) Tumor growth curve of nude mice in the indicated two groups. (**D**) The statistical results of the weight of tumors. (**E**) Immuno -histochemistry staining of xenograft model-derived tumors for Ki67and FSP1. Scale bar = 20 μm. Data are expressed as the mean ± SD. *P < 0.05, **P < 0.01

The in vivo study was carried out to validate the dataset outcomes obtained from in vitro experiments. U87 cell lines were stably transfected with Scramble-shRNA or hLINC02587-shRNA and subcutaneously injected into nude murines. The dataset outcomes showed that suppressing LINC02587 greatly reduced tumor development (Fig. 7A, B). As demonstrated in Fig. 7C, and D, the volume and weight of tumors in the LINC02587 silencing cohort were much more reduced than in the empty vector cohort. Furthermore, IHC staining demonstrated that LINC02587 silencing decreased the expressions of Ki67 and FSP1 (Fig. 7E). Taken together, the findings validated LINC02587’s tumor-promoting activity in glioma.

## Discussion

Over the past few decades, numerous studies have demonstrated that some functional lncRNAs are down-regulated in a variety of cancers [18, 19]. Due to their roles in regulating tumor proliferative and metastasis, they have been suggested as potential therapeutic targets and biomarkers [20, 21]. It is thought that glioma tissue has an elevated level of lncRNAs that are misaligned and implicated within the control of the glioma cell cycle, proliferative / differentiation / apoptotic activities, and other cellular events [6, 22]. High LINC02587 (also called MEOX2-AS1) expression has previously been seen in breast cancer and colon adenocarcinoma [14, 15]. These dataset outcomes demonstrate the potential significance of LINC02587 in the development of tumors. In this view, this study aimed to examine whether LINC02587 serves as a functional regulator in gliomas. According to the obtained dataset outcomes, LINC02587 expression is elevated within glioma tissue and cell lines and it is favorably connected with the clinical stage in such oncology cases. Both GEPIA and RT-qPCR confirmed this result. Evaluation of survival data identified that LINC02587 upregulation was linked to poor prognosis, suggesting that the underlined gene could serve as a novel biomarker in glioma. For the functional cellular tests, the siRNAs were generated that specifically target LINC02587 and transfected them into glioma cell lines. Silencing LINC02587 inhibited cell proliferative, migrative, and invasive while increasing cell death in glioma cells.

EMT is a pervasive biological process and a crucial step in cancer and progression metastasis [23]. EMT is a typical biological process that is essential for the development of cancer metastasis. It is characterized by epithelial cells acquiring a mesenchymal phenotype. The increase in invasive and motility made it even more severe. Herein, the LINC02587 silencing decreased the protein expression of MMP2, N-cadherin, ZEB1, and ZO1, indicating that LINC00886 may be associated with the EMT procedure.

Based on the above experimental dataset outcomes, the likely upstream and downstream regulatory mechanisms of LINC02587 were examined. Epigenetic modifications, particularly DNA methylation, are widely considered to govern tumor growth and the development of secondary malignant growth [10, 24]. During the current evaluation, the UCSC Genome Browser revealed 4 CpG islands within the LINC02587 promoter regions. Consequently, this study hypothesized that DNA methylation may impact LINC02587 expression. First, we saw that 5-Aza-dC treatment considerably raised the expression of LINC02587 in LN229 or U87 cells and that this increase was dose-dependent. These findings indicate that DNA methylation could be a contributor to upstream regulatory mechanisms of LINC02587 expression. The promoter CpG island’s aberrant methylation state was confirmed by BSP and MSP. These findings helped in understanding the role of DNA methylation in the development of glioma carcinogenesis and provided a novel perspective on the origins of the LINC02587 mutation. Furthermore, high-throughput sequencing technology was employed to conduct a detailed examination of downstream target genes and signaling pathways where LINC02587 could play a part.

Such findings revealed that FSP1 (also known as AIFM2) was closely associated with LINC02587. Previous investigations demonstrated the CoQ-FSP1 pathway to be associated with regulating ferroptosis [25–27]. Ferroptosis is a type of controlled cell death that is characterized by lipid peroxidation which is an important factor in the initiation of ferroptosis. Tumor cells, on the other hand, frequently alter their metabolic pathway to maintain redox equilibrium, which leads to ferroptosis resistance. Interfering with the essential metabolic process associated with intracellular redox equilibrium may therefore increase the therapeutic efficacy of ferroptosis. As an oxidoreductase, ferroptosis suppressor protein 1 (FSP1) can convert ubiquinone (CoQ) to ubiquinol (CoQH_2_) primarily on the plasma membrane. CoQH_2_ works as an antioxidant, reducing lipophilic radicals and maintaining intracellular redox equilibrium, resulting in ferroptosis resistance. The CoQ/FSP1 axis is responsible for establishing the correlation between ferroptosis and the metabolic pathway. In this view, the current study hypothesized that LINC02587 may be associated with ferroptosis. According to the dataset outcomes obtained from the western blot, LINC02587 silencing may cause a drop in the levels of CoQ10B and FSP1.

- Herein, compared to the si-NC cohort, LINC02587 deficiency was associated with increased iron levels and lipid peroxidation, as well as decreased mitochondrial membrane potential. These dataset outcomes indicate that silencing LINC02587 may induce oxidative stress-related ferroptosis in glioma cells in vitro or that it can promote ferroptosis in glioma cells.
- Following that, in vivo assays were performed using a xenograft model developed by subcutaneously injecting U87 cells infected with hLINC02587-shRNA into nude murines. According to the obtained dataset outcomes, inhibiting LINC02587 could inhibit glioma growth in vivo.

## Conclusion

**Fig. 8:**
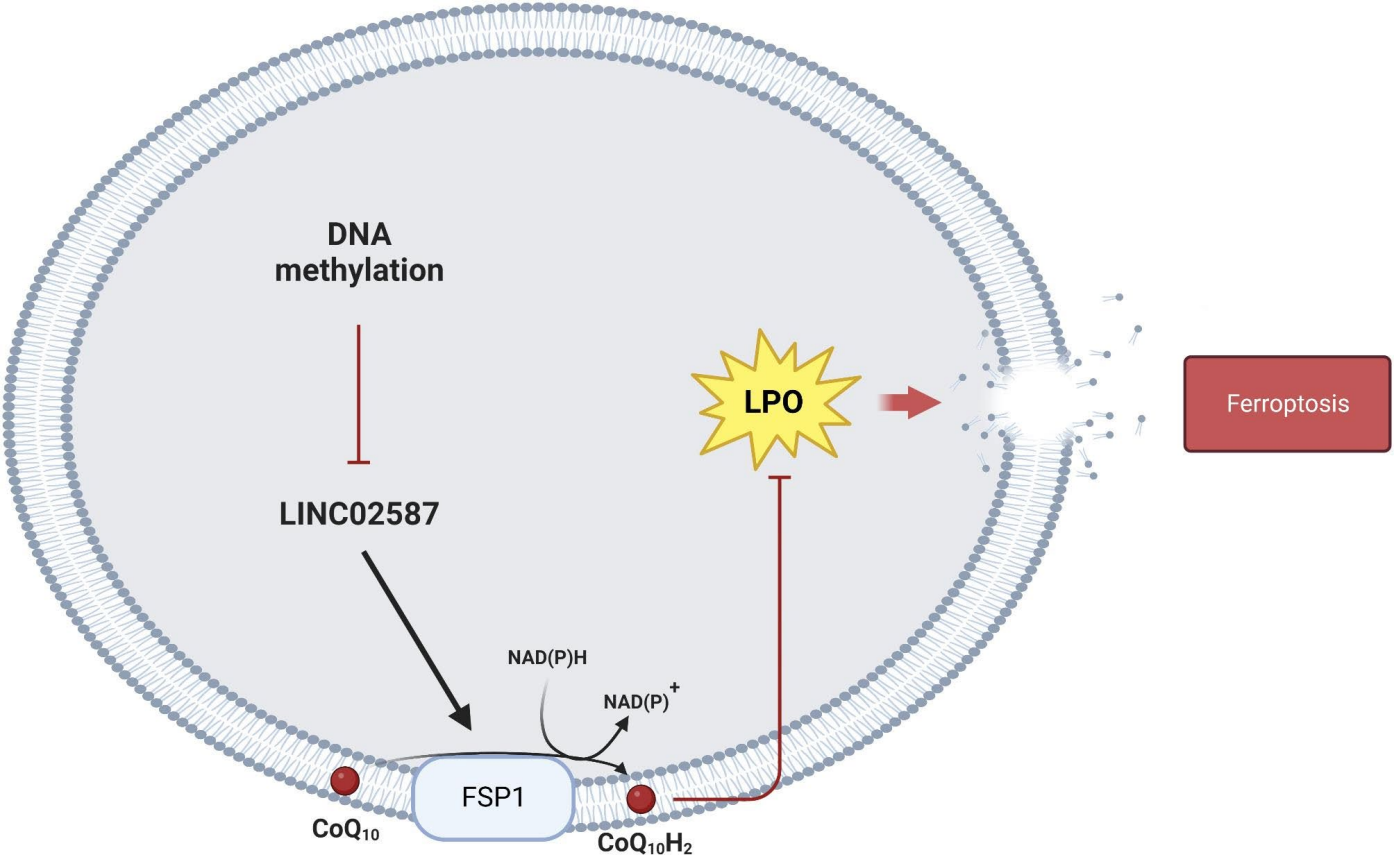
The schematic diagram of the roles of LINC02587 in glioma ferroptosis.The expression of LINC02587 in glioma cells is regulated by DNA methylation

Taken together, altering the methylation state of CpG islands in LINC02587’s upstream promoter regions can change the expression level of the gene. Silencing LINC02587 can increase the incidence of ferroptosis in glioma cells via the CoQ/FSP1 axis, inhibiting tumor proliferative(Fig. 8). Herein, this study revealed the molecular basis for how LINC02587 functions in gliomas and proposes that it could be employed as a novel prognostic biomarker and therapeutic target for glioma patients. A detailed investigation on LINC02587 will be carried out in one of our upcoming research.

### Electronic supplementary material

Below is the link to the electronic supplementary material.

Supplementary Material 1

Supplementary Material 2

## Data Availability

The datasets used in the current study are openly available in UCSC (http://genome.ucsc.edu/), and GEPIA (http://gepia.cancer-pku.cn/index. html). All date and materials are available from the corresponding authors upon request.
