# Supplementary material for: DNA methylation-regulated LINC02587 inhibits ferroptosis and promotes the progression of glioma cells through the CoQ-FSP1 pathway
Source: BMC Cancer. 2023 Oct 17;23:989. doi: 10.1186/s12885-023-11502-0 (PMC10580646; doi:10.1186/s12885-023-11502-0)
Supplement: Supplementary file 1 — Supplementary Material 1 [file 12885_2023_11502_MOESM1_ESM.docx]

**Table.S1 Primer names and sequences**

| **Gene** | **Sequence（5’ to 3’）** |
| --- | --- |
| LINC02587-F | TCCATAATCCTCTCCACCACCAGTC |
| LINC02587-R | CAAGTGCTCAACAACCACACGAATC |
| GAPDH-F | GGAGCGAGATCCCTCCAAAAT |
| GAPDH-R | GGCTGTTGTCATACTTCTCATGG |
| U6-F | TGCGGGTGCTCGCTTCGGCAGC |
| U6-R | CCAGTGCAGGGTCCGAGGT |
| Negative control-F | UUCUCCGAACGUGUCACGUTT |
| Negative control-R | ACGUGACACGUUCGGAGAATT |
| si-LINC02587-i-F | GCAAGUCUGACGGUACCAATT |
| si-LINC02587-i-R | UUGGUACCGUCAGACUUGCTT |
| si-LINC02587-ii-F | GGGUCACAUGCCUGGUGUUTT |
| si-LINC02587-ii-R | AACACCAGGCAUGUGACCCTT |
| si-LINC02587-iii-F | GCUCAUGGACCAGAACAAATT |
| si-LINC02587-iii-R | UUUGUUCUGGUCCAUGAGCTT |
| BSP primer（LN229）： |  |
| Linc02587-1F | GGTTGGTTTAGGAGTTTAGAGTTGA |
| Linc02587-1R | CTTTTCTACAAATAACAATTCCCAA |
| Linc02587-1FO | TGTTAAGAGTGGGAGAGGTTGAA |
| Linc02587-1RO | TCCCAACCTATTAACAACCCAC |
| MSPprimer（LN229）： |  |
| Linc02587-1MF | TGGACGGACGTAGAGCGTTTAC |
| Linc02587-1MR | CCCGAACGAAACTTAAACGTTAA |
| Linc02587-1UF | TGTTGGATGGATGTAGAGTGTTTAT |
| Linc02587-1UR | CCTCCCAAACAAAACTTAAACATTAA |
| BSP primer（U87）： |  |
| Linc02587-2F | TGGGAATTGTTATTTGTAGAAAAGT |
| Linc02587-2R | AAACATCTACAACCCTTTCAAACTC |
| Linc02587-2FO | TYGGGAGGGTAGTAGTTTTGTT |
| Linc02587-2RO | CACATCTCATATAACCRAAACACA |
| MSP primer（U87）： |  |
| Linc02587-2MF | TGTATTTTTTCGTGTTTCGGGC |
| Linc02587-2MR | CCGCAACCTCAAATCTAACGTC |
| Linc02587-2UF | TTTTGTATTTTTTTGTGTTTTGGGT |
| Linc02587-2UR | CCCCACAACCTCAAATCTAACATC |
| Genes associated with ferroptosis: |  |
| STEAP3-F | AATGAGAGGCAGGGAGAGC |
| STEAP3-R | CTTCAGCCAGAGGTGGGT |
| FTH1-F | AGTCGTCGGGGTTTCCT |
| FTH1-R | GAGGGTGCGGTGAAGAG |
| ACSL5-F | GGCATTGGTGCTGATAGG |
| ACSL5-R | TCTTCTCCCCTCTTTGCTT |
| ATG5-F | GCTTCGAGATGTGTGGTTT |
| ATG5-R | GTTCTGCTTCCCTTTCAGTT |
| SLC39A14-F | GTGTGGGGCTTTGGTTT |
| SLC39A14-R | CAGGGCGATGAAGTAAGTG |
| HMOX1-F | GTGACCCGAGACGGCTT |
| HMOX1-R | ACAGGGGCGAAGACTGG |
| F3-F | TGGCACCTTTTGCACATA |
| F3-R | TTTGCTTGGACGACCTG |
| SLC3A2-F | ATCAAGGTGGCGGAAGAC |
| SLC3A2-R | CAGCCGAGCCAGAAGAG |
| AIFM2-F | ACAGCCAGCCCTTCCTC |
| AIFM2-R | TGCCCTCACAGACAGACAC |
| ACSL3-F | CAAGCTGAAACGCAAAGAG |
| ACSL3-R | CAAACTGATGCCAGAAGAGA |
| ACSL1-F | ACACACAGGGGACATTGG |
| ACSL1-R | AACAGGCTCACTTCGCAT |
| PRNP-F | CTGCTGGATGCTGGTTCT |
| PRNP-R | GAGGTGGGTAGCGGTTG |

**Table.S2 CpG island sequences**

| Region 1 | GGCTGGCTTAGGAGCCCAGAGTTGACCACATGCAAACTCCCTCCAAAACTCCCGCTCAGCCTGCGCCACGCGCTCCCAGTTCCACTTCCTATCTCCTGCTGGACGGACGTAGAGCGTCCACGCCCGCCCAGCACTGCCGGCTGCCAGGACTACAAGCGGCACGCCAACGTCTAAGTCCCGCCCGGGAGGGCAGCAGCTCTGCCCTCCAGACTCTGGAGGCCTGGGCGCGGCGCACACCACCCCCAACCTAGCACCCTGATTCCTACCTACTCACTTTCCTCTCGGGGAAACGTGACTTGTCAGTGTCATTTGGGAACTGTCACCTGCAGAAAAG |
| --- | --- |
| Region 2 | TGGGAACTGTCACCTGCAGAAAAGCTAGGGATACGCCTATCCTTTCAGGAACACGAAAGAAGTAATAGGGTTAAAGGAAAAGACCAGAGCTAGTTTGGCGCACCCTCGAAGTTTTTCAAACTTTGCATCTTCCCGTGCCCCGGGCGCCGCAGCGCGCCTAGGTTTTCGCAGCTGGTTTCTGTCGGTGGGTTGTTAATAGGCTGGGACGCCAGATCTGAGGTTGCGGGGATGAGCACCATGGCCTGGAAAAGGCGGTTCTCAGTGCAGAAAGAAGACGGACCTGGTTGGGTGGGTGGGAGGAAGGCGTGAGTCTGAAAGGGTTGCAGATGCCT |
