# Supplementary material for: DNA methylation-regulated LINC02587 inhibits ferroptosis and promotes the progression of glioma cells through the CoQ-FSP1 pathway
Source: BMC Cancer. 2023 Oct 17;23:989. doi: 10.1186/s12885-023-11502-0 (PMC10580646; doi:10.1186/s12885-023-11502-0)
Supplement: Supplementary file 2 — Supplementary Material 2 [file 12885_2023_11502_MOESM2_ESM.pdf]

Supplementary figures 1

cells+PCR

	229	A172	SNB19	T98	U87	U251
LINC02587	22.77	18.43	25.73	21.8	18.84	25.59
LINC02587	22.48	19.87	26.39	21.87	19.34	25.51
LINC02587	22.54	19.61	25.97	21.75	19.06	25.33
GAPDH	9.79	8.88	11.99	11.73	9.81	12.91
GAPDH	9.63	8.8	11.99	11.67	9.79	12.98
GAPDH(平均	9.71	8.84	11.99	11.7	9.8	12.945
	-13.06	-9.59	-13.74	-10.1	-9.04	-12.645
	-12.77	-11.03	-14.4	-10.17	-9.54	-12.565
	-12.83	-10.77	-13.98	-10.05	-9.26	-12.385
	0.000117	0.001298	7.31E-05	0.000911	0.0019	0.000156
	0.000143	0.000478	4.63E-05	0.000868	0.001343	0.000165
	0.000137	0.000573	6.19E-05	0.000943	0.001631	0.000187
	1.60214	17.75311	1	12.46663	25.99208	2.136131
	3.09513	10.33882	1	18.76536	29.04061	3.567714
	2.219139	9.253505	1	15.24221	26.35491	3.020945

Nucleoplasmic separation assay+RCR

LN229	C质	C质	C质	C核	C核	C核
gapdh	12.69	12.81	12.72	14.76	14.71	14.65
u6	29.35	29.24	29.3	28.33	28.45	28.52
linc02587	22.95	23.27	23.18	25.39	25.56	25.71
比例	C质	C质	C质	C核	C核	C核
gapdh	0.80765	0.788679	0.792124	0.19235	0.211321	0.207876
u6	0.33026	0.366425	0.368035	0.66974	0.633575	0.631965
linc02587	0.844392	0.830237	0.852414	0.155608	0.169763	0.147586

U87	C质	C质	C质	C核	C核	C核
gapdh	12.48	12.61	12.42	14.65	14.86	14.96
u6	29.07	29.3	29.15	27.95	28.29	28.12
linc02587	23.42	23.67	23.33	25.78	25.65	25.84
	C质	C质	C质	C核	C核	C核
	0.81819	0.826293	0.853284	0.18181	0.173707	0.146716
	0.315113	0.331795	0.328728	0.684887	0.668205	0.671272
	0.836966	0.797773	0.850662	0.163034	0.202227	0.149338

Tissue+PCR

linc02587 (NC)	28.72	30.88	30.92	28.13	29.74	28.68
linc02587 (NC)	28.22	30.85	30.86	28.1	29.94	28.64
AVERAGE	28.47	30.865	30.89	28.115	29.84	28.66
gapdh (NC)	16.24	18.68	16.58	15.84	16.74	15.52
gapdh (NC)	16.43	18.61	16.66	15.85	16.8	15.53
AVERAGE	16.335	18.645	16.62	15.845	16.77	15.525
△CT	-12.135	-12.22	-14.27	-12.27	-13.07	-13.135
2^△CT	0.000222	0.00021	5.06E-05	0.000202	0.000116	0.000111
con平均	0.000152					
结果	1.46193	1.378285	0.332834	1.331335	0.764651	0.730965

linc02587(Ⅰ)	24.68	26.31	24.87	26.61	26.29	27.69	24.31	25.67	26.27
linc02587(Ⅰ)	24.34	26.27	24.62	26.51	28.88	27.65	24.45	25.94	26.13
	24.51	26.29	24.745	26.56	27.585	27.67	24.38	25.805	26.2
gapdh(Ⅰ)	15.6	15.72	15.72	15.24	10.88	18.63	13.48	17.26	16.42
gapdh(Ⅰ)	15.48	15.59	15.63	14.92	10.61	17.6	13.47	19.7	16.24
	15.54	15.655	15.675	15.08	10.745	18.115	13.475	18.48	16.33
	-8.97	-10.635	-9.07	-11.48	-16.84	-9.555	-10.905	-7.325	-9.87
	0.001994	0.000629	0.001861	0.00035	8.52E-06	0.001329	0.000522	0.006237	0.001069

linc02587 (II)	22.81	26.56	25.78	23.56	24.97	24.34	24.67	25.77	24
linc02587 (II)	22.56	26.29	25.77	23.44	24.98	23.93	24.63	25.55	24.27
	22.685	26.425	25.775	23.5	24.975	24.135	24.65	25.66	24.135
gapdh (II)	15.59	16.89	16.56	16.46	17.24	16.53	15.68	17.59	15.9
gapdh (II)	15.66	16.8	16.6	16.55	17.29	15.58	15.7	17.55	15.86
	15.625	16.845	16.58	16.505	17.265	16.055	15.69	17.57	15.88
	-7.06	-9.58	-9.195	-6.995	-7.71	-8.08	-8.96	-8.09	-8.255
	0.007494	0.001307	0.001706	0.00784	0.004776	0.003696	0.002008	0.00367	0.0032734
	49.27808	8.591285	11.21901	51.54905	31.40395	24.29982	13.20373	24.13197	21.523996

linc02587(III)	25.91	25.37	28.62	23.56	24.95	23.98	25.53	29.49	25.47	30.21
linc02587(III)	25.75	25.87	29.47	23.85	24.72	23.8	26.26	29.52	25.78	29.72
	25.83	25.62	29.045	23.705	24.835	23.89	25.895	29.505	25.625	29.965
gapdh(III)	16.72	18.26	22.48	15.44	15.83	16.47	18.57	22.44	17.88	20.91
gapdh(III)	16.84	18.19	22.51	15.67	15.7	16.71	18.47	20.48	17.93	21.16
	16.78	18.225	22.495	15.555	15.765	16.59	18.52	21.46	17.905	21.035
	-9.05	-7.395	-6.55	-8.15	-9.07	-7.3	-7.375	-8.045	-7.72	-8.93
	0.001886594	0.005941	0.010672	0.003521	0.001861	0.006346	0.006024	0.003786	0.004743	0.00205
	12.40520795	39.06688	70.17445	23.14894	12.23442	41.72598	39.61223	24.89655	31.18702	13.48117

linc02587(IV)	22.86	24.77	25.51	24.72	27.49	24.5	26.45	24	24.27	26.46
linc02587(IV)	24.86	24.78	25.48	24.43	27.58	25.08	26.46	24.62	24.83	26.21
	23.86	24.775	25.495	24.575	27.535	24.79	26.455	24.31	24.55	26.335
gapdh(IV)	16.78	15.96	18.55	18.56	19.38	16.39	19.26	15.82	18.46	18.49
gapdh(IV)	16.83	15	18.6	18.88	19.5	16.52	19.48	15.98	18.51	18.28
	16.805	15.48	18.575	18.72	19.44	16.455	19.37	15.9	18.485	18.385

Normal	Grade (I	Grade (III+IV)
1.461929778	13.11253	12.40521
1.378284787	4.134962	39.06688
0.332833837	12.23442	70.17445
1.331335347	2.301978	23.14894
0.764651362	0.056051	12.23442
0.730964889	8.741459	41.72598
	3.429205	39.61223
	41.00915	24.89655
	7.02683	31.18702
	49.27808	13.48117
	8.591285	49.44916
	11.21901	10.46771
	51.54905	54.29976
	31.40395	113.6043
	24.29982	24.04848
	13.20373	20.36294
	24.13197	48.43151
	21.524	19.3314
		98.21517
		26.59115

Supplementary figures 2

methylation+PCR

229	control	5A-5um	5A-10um	u87		control	5A-5um	5A-10um
linc02587	37.33	33.96	33.9		linc02587	32.78	32.39	31.42
linc02587	37.17	34.91	33.98		linc02587	32.89	31.88	30.81
linc02587	34.87	33.09	32.78		linc02587	33.87	32.69	31.48
GAPDH	14.1	13.55	14.68		GAPDH	13.14	14.54	13.79
GAPDH	14.25	13.62	14.29		GAPDH	13.01	14.75	13.79
GAPDH(平均)	14.175	13.585	14.485			13.075	14.645	13.79
	-23.155	-20.375	-19.415			-19.705	-17.745	-17.63
	-22.995	-21.325	-19.495			-19.815	-17.235	-17.02
	-20.695	-19.505	-18.295			-20.795	-18.045	-17.69
	1.07066E-07	7.35383E-07	1.43055E-06			1.17005E-06	4.55221E-06	4.92993E-06
	1.19623E-07	3.80658E-07	1.35338E-06			1.08415E-06	6.48258E-06	7.52436E-06
	5.89094E-07	1.34403E-06	3.10925E-06			5.49644E-07	3.69755E-06	4.72911E-06
mean	2.71927E-07					9.34615E-07		
	0.393728978	2.704336736	5.260773012			1.251904232	4.870683378	5.274828553
	0.439908323	1.399852483	4.976994536			1.159999516	6.936097618	8.050755655
	2.166362698	4.942615924	11.43413087			0.588096253	3.956224246	5.059953767

agarose gel electrophoresis

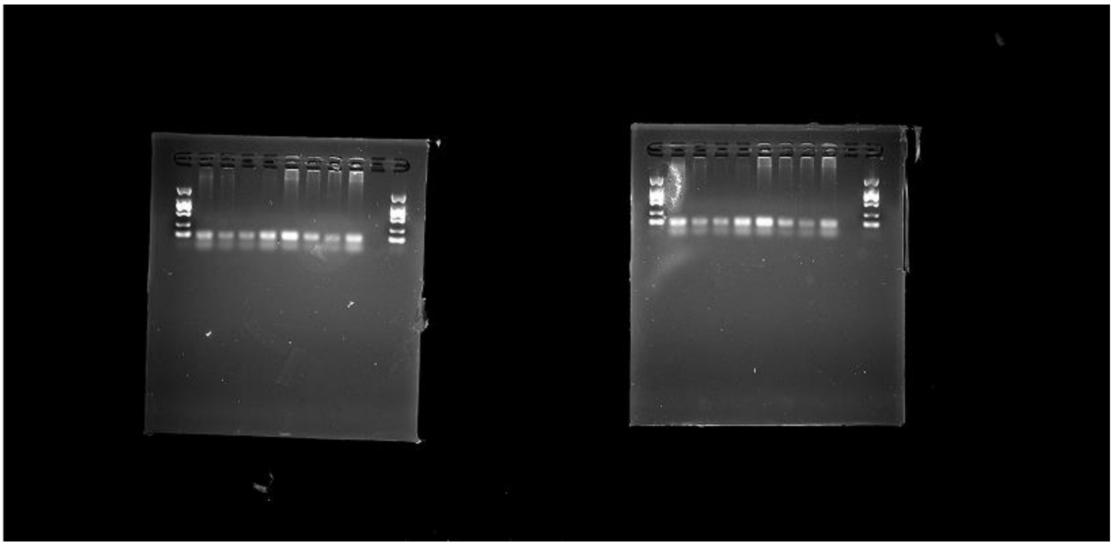

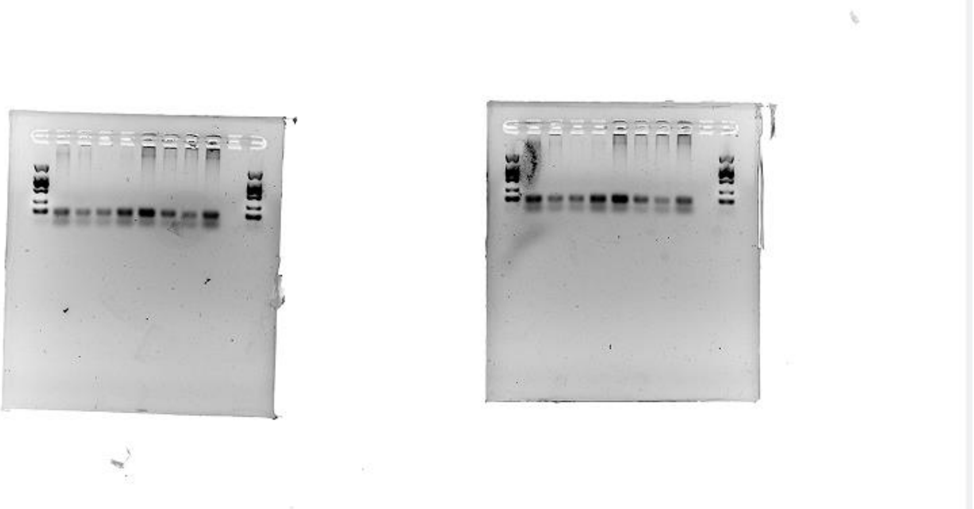

Supplementary figures 3

CCK8+229

229	NC	NC	NC	SI-2	SI-2	SI-2	SI-3	SI-3	SI-3
0	0. 2419	0. 2426	0. 2423	0. 2424	0. 243	0. 2424	0. 2422	0. 2416	0. 2417
24	0. 8055	0. 8347	0. 8875	0. 6665	0. 628	0. 6943	0. 7279	0. 6984	0. 7001
48	1. 7641	1. 7387	1. 7324	1. 4776	1. 5495	1. 4548	1. 5513	1. 5046	1. 5274
72	2. 2641	2. 2387	2. 3324	1. 6776	1. 6495	1. 6548	1. 6513	1. 6046	1. 6274

CCK8+u87

u87	NC	NC	NC	SI-2	SI-2	SI-2	SI-3	SI-3	SI-3
0	0. 2399	0. 2351	0. 2308	0. 2339	0. 2383	0. 2346	0. 2373	0. 2352	0. 2358
24	0. 7914	0. 7043	0. 7532	0. 6808	0. 6257	0. 6753	0. 6763	0. 7013	0. 6982
48	1. 725	1. 719	1. 793	1. 4837	1. 4064	1. 4652	1. 4345	1. 4293	1. 4487
72	2. 1028	2. 1056	2. 1231	1. 6477	1. 6731	1. 6322	1. 7012	1. 7013	1. 7651

flow cytometry+apoptosis

	NC	si-1	si-2
	4.35	10. 75	11. 17
229+02587	6. 2	10. 1	11. 3
	7. 97	11.05	11. 89
	16. 46	24. 22	25. 13
U87+02587	17. 5	24. 39	26. 31
	17. 97	24.47	28. 27

COLONY									
229									
	NC	NC	NC	-LINC02587	-LINC02587	-LINC02587	-LINC02587-	-LINC02587-	-LINC02587-iii
	202	189	178	94	109	82	82	71	69
87									
	88	73	65	58	49	42	45	56	47

transfection efficiency+PCR

229	NC1	L1	L2	L3
LINC0258	31.63	35.03	34.45	34.87
LINC0258	32.73	35.74	34.56	35.56
LINC0258	32.26	34.68	34.89	34.91
GAPDH	14.96	17.11	15.15	16.59
GAPDH	15.01	17.06	15.2	16.59
GAPDH(平	14.985	17.085	15.175	16.59
	-16.645	-17.945	-19.275	-18.28
	-17.745	-18.655	-19.385	-18.97
	-17.275	-17.595	-19.715	-18.32
	9.75789E-06	3.96293E-06	1.57633E-06	3.14175E-06
	4.55221E-06	2.42262E-06	1.46061E-06	1.94743E-06
	6.30532E-06	5.051E-06	1.16197E-06	3.05584E-06
mean	6.87181E-06			
	1.419988671	0.576694601	0.229390797	0.457194331
	0.662448139	0.352545023	0.212550774	0.283393666
	0.91756319	0.735032232	0.169091896	0.444692328

U87	NC	L12	L22	L32
LINC02587	31.75	33.43	31.82	30.73
LINC02587	32.48	33.21	31.19	31.65
LINC02587	31.96	33.06	31.57	31.24
GAPDH	17.71	17.88	14.6	14.09
GAPDH	17.68	17.87	14.55	14.13
GAPDH(平均值	17.695	17.875	14.575	14.11
	-14.055	-15.555	-17.245	-16.62
	-14.785	-15.335	-16.615	-17.54
	-14.265	-15.185	-16.995	-17.13
	5.87521E-05	2.0772E-05	6.4378E-06	9.92845E-06
	3.54219E-05	2.41939E-05	9.96292E-06	5.24728E-06
	5.07934E-05	2.68448E-05	7.65588E-06	6.97198E-06
mean	4.83225E-05			
	1.215834471	0.4298624	0.133225932	0.205462474
	0.733031361	0.500675174	0.206175788	0.108588771
	1.051134167	0.555533889	0.158433226	0.144280359

WB

229												
	Normal	Normal	Normal	si-NC	si-NC	si-NC	si-LINC02	si-LINC02	si-LINC02	si-LINC02	si-LINC02	si-LINC02587-iii
Bcl-2	20982.33	19131.1	22965.06	22051.43	20596.83	22632.96	11534.89	12043.23	12110.93	14758.77	15374	16584.49
Bax	13779.96	11216.73	13405.82	13375.55	13507.03	12071.58	29254.96	28347.34	26079.31	28184.92	28883.72	28961.78
actin	21825.34	20827.28	22738.67	23448.33	22152.5	21559.96	26848.59	25081.72	23280.63	26852.3	26489.65	26332.53
Bcl-2/act	0.961375	0.91856	1.009956	0.940426	0.929774	1.049768	0.429628	0.480159	0.520215	0.549628	0.580378	0.62981
Bax/actir	0.631375	0.53856	0.58956	0.570426	0.609729	0.559907	1.089628	1.130199	1.120215	1.049628	1.090378	1.099848
87												
	Normal	Normal	Normal	si-NC	si-NC	si-NC	si-LINC02	si-LINC02	si-LINC02	si-LINC02	si-LINC02	si-LINC02587-iii
Bcl-2	22139.14	21498.16	23019.07	23982.26	23869.93	24624.63	13282.32	12397.34	9812.874	13199.88	12109.25	11412.7
Bax	21910.79	19761.34	23921.58	23267.81	22710.95	26133.57	28073.87	29341.96	25474.72	29767.17	31492.06	30009.65
actin	22834.16	21710.26	22562.81	24481.9	23179.54	25132.28	25065.42	25290.48	23375.89	25880.15	26920.56	26552.79
Bcl-2/act	0.969562	0.99023	1.020222	0.979592	1.029784	0.979801	0.529906	0.490198	0.419786	0.510039	0.449814	0.429812
Bax/actir	0.959562	0.91023	1.060222	0.950408	0.979784	1.039841	1.120024	1.160198	1.089786	1.150193	1.169814	1.130188

LN229

replicate blots 1 replicate blots 2 replicate blots 3

26kDa

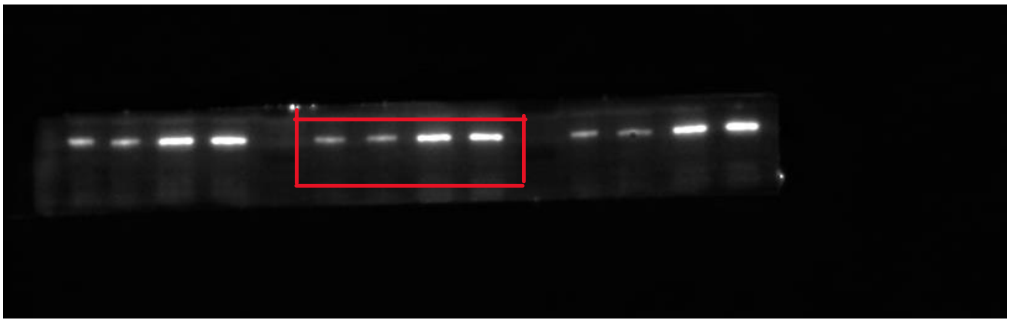

Bax

replicate blots 1 replicate blots 2 replicate blots 3

26kDa

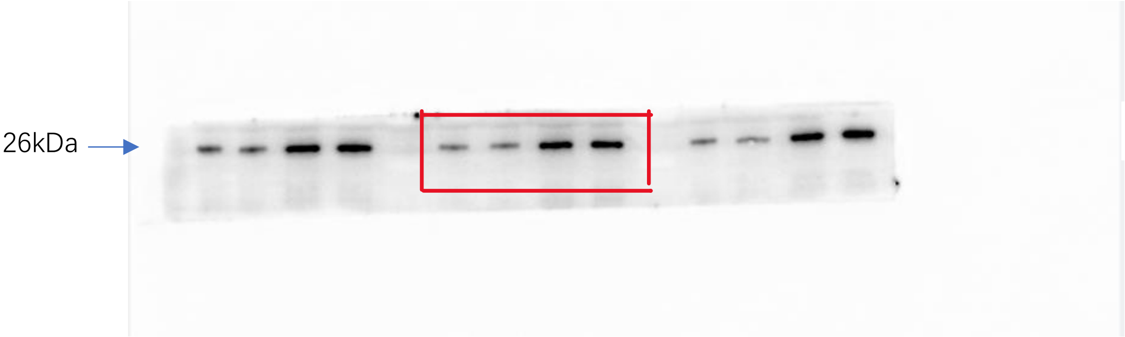

Bax

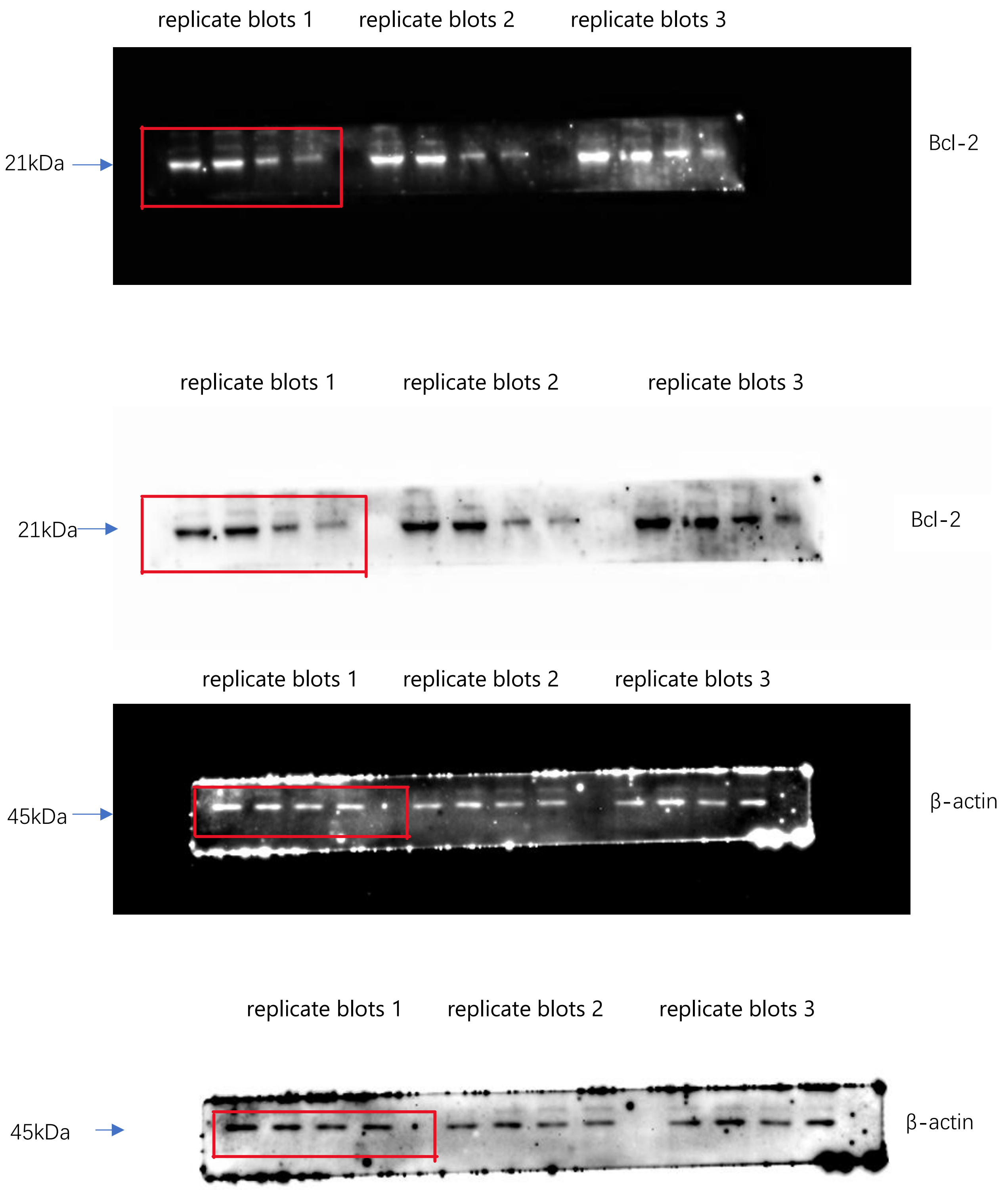
replicate blots 1
replicate blots 2
replicate blots 3
21kDa
Bcl-2
replicate blots 1
replicate blots 2
replicate blots 3
21kDa
Bcl-2
replicate blots 1
replicate blots 2
replicate blots 3
45kDa
β-actin
replicate blots 1
replicate blots 2
replicate blots 3
45kDa
β-actin

U87

replicate blots 1 replicate blots 2 replicate blots 3

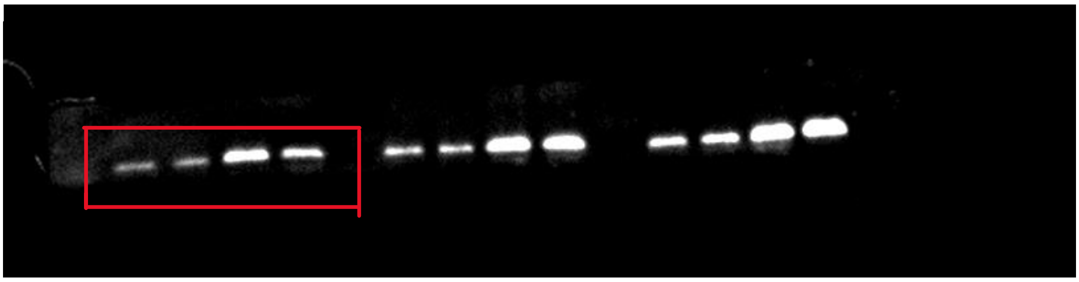

26kDa → Bax

replicate blots 1 replicate blots 2 replicate blots 3

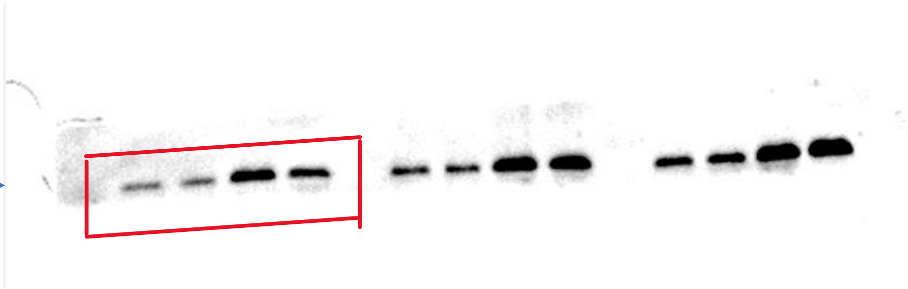

26kDa → Bax

replicate blots 1 replicate blots 2 replicate blots 3

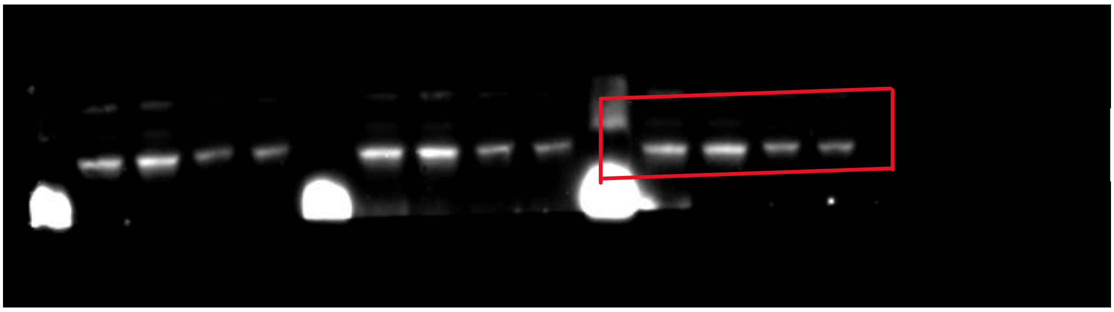

21kDa → Bcl-2

replicate blots 1 replicate blots 2 replicate blots 3

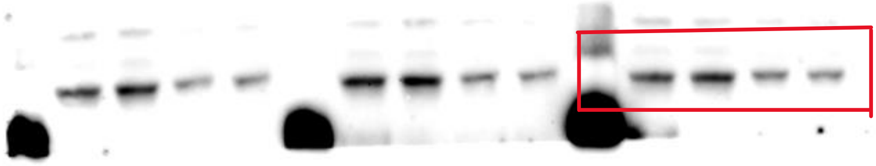

21kDa → Bcl-2

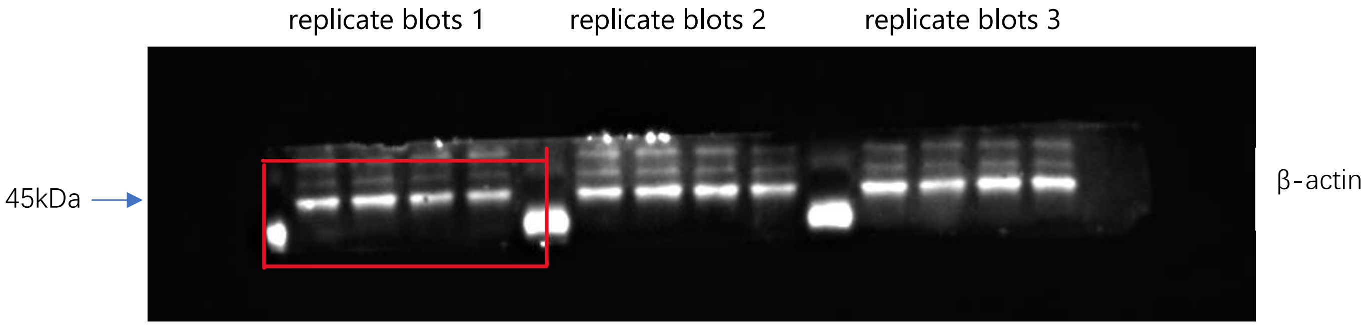
replicate blots 1
replicate blots 2
replicate blots 3
45kDa
β-actin

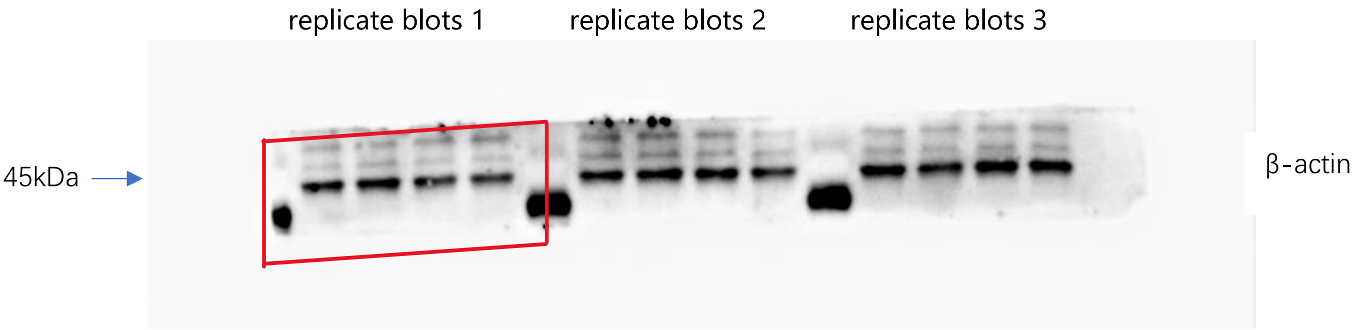
replicate blots 1
replicate blots 2
replicate blots 3
45kDa
β-actin

Supplementary figures 4

wound healing									
229									
	NC	NC	NC	-LINC02587	-LINC02587	-LINC02587	-LINC02587-	-LINC02587-	-LINC02587-iii
	45. 12	43. 25	39. 37	23. 21	21. 35	25. 19	29. 38	24. 67	23. 85
87	52. 57	48. 21	54. 35	24. 63	22. 19	26. 34	28. 92	27. 52	25. 98
transwell									
	NC	NC	NC	-LINC02587	-LINC02587	-LINC02587	-LINC02587-	-LINC02587-	-LINC02587-iii
229	142	128	113	85	89	95	76	65	82
87	97	92	106	52	59	61	62	75	68

transwell image(LN229)

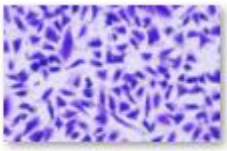
tanswell-229-NC1

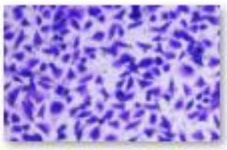
tanswell-229-NC2

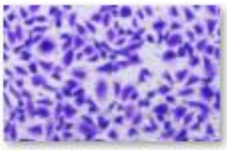
tanswell-229-NC3

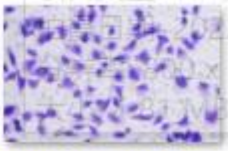
tanswell-229-si-ii-1

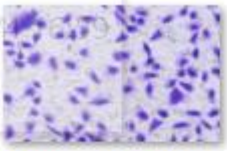
tanswell-229-si-ii-2

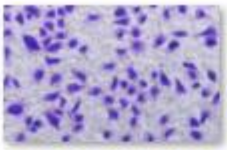
tanswell-229-si-ii-3

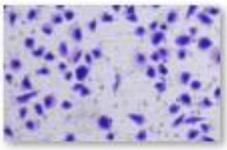
tanswell-229-si-iii-1

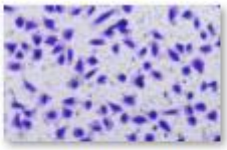
tanswell-229-si-iii-2

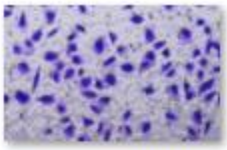
tanswell-229-si-iii-3

transwell image(U87)

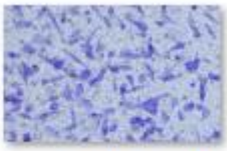
tanswell-87-NC1

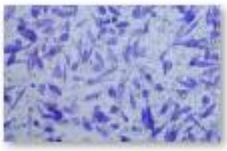
tanswell-87-NC2

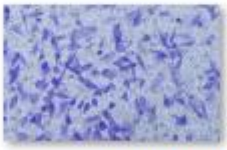
tanswell-87-NC3

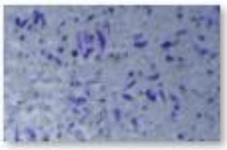
tanswell-87-si-iii-1

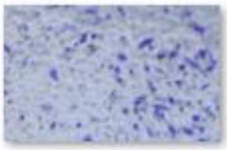
tanswell-87-si-iii-2

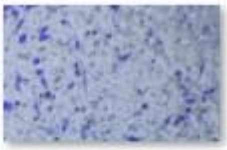
tanswell-87-si-iii-3

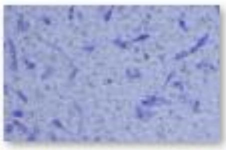
tanswell-87-si-ii-1

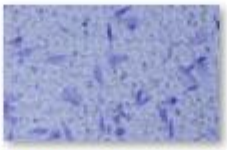
tanswell-87-si-ii-2

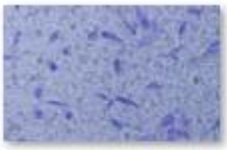
tanswell-87-si-ii-3

wound healing image(LN229-0h)

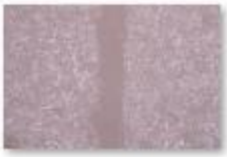
NC-1

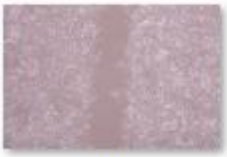
NC-2

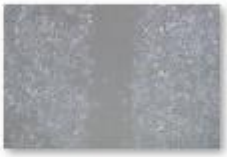
NC-3

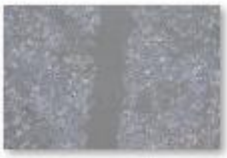
si-ii-1

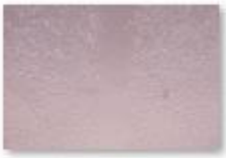
si-ii-2

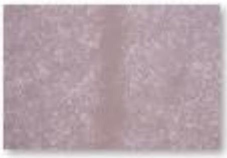
si-ii-3

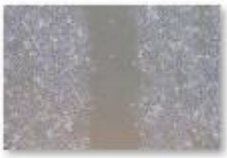
si-iii-1

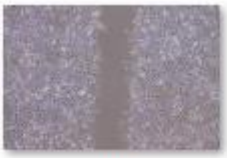
si-iii-2

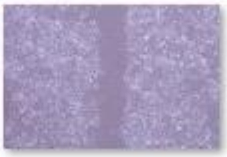
si-iii-3

wound healing image(LN229-24h)

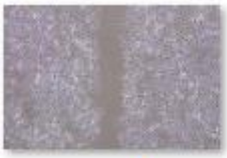
nc-1

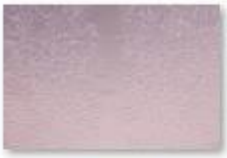
nc-2

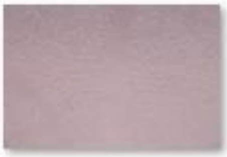
nc-3

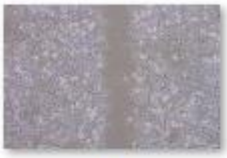
si-ii-1
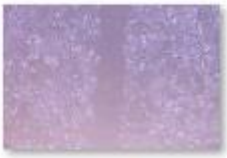
si-ii-2
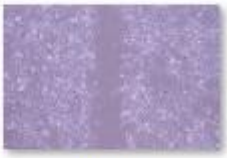
si-ii-3
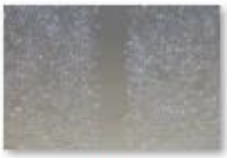
si-iii-1
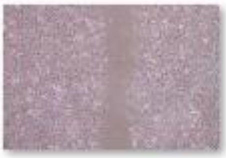
si-iii-2
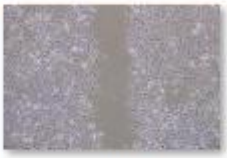
si-iii-3

wound healing image(U87-0h)

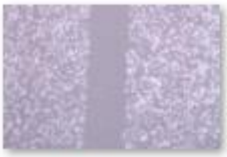
NC-1
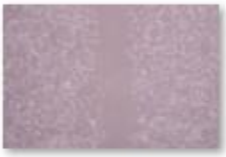
NC-2
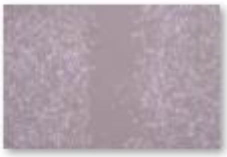
NC-3
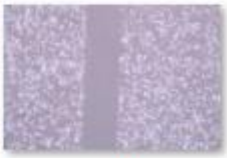
si-ii-1
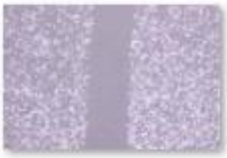
si-ii-2
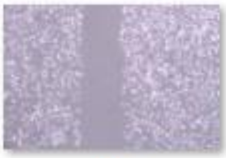
si-ii-3
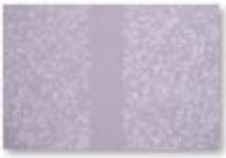
si-iii-1
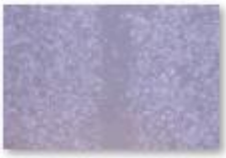
si-iii-2
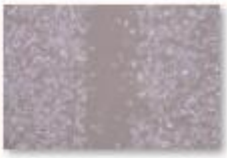
si-iii-3

wound healing image(U87-24h)

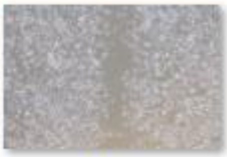
NC-1
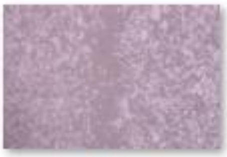
NC-2
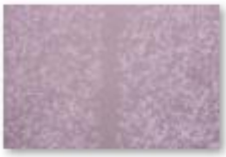
NC-3

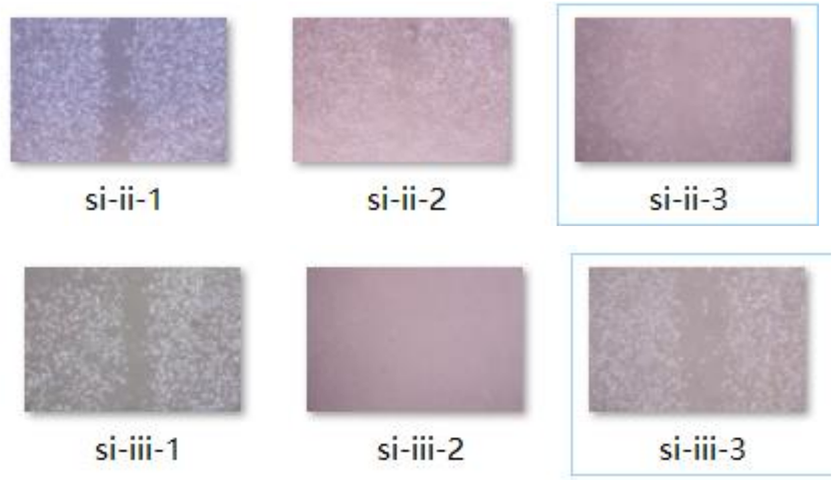

229												
	Normal	Normal	Normal	si-NC	si-NC	si-NC	si-LINC02	si-LINC02	si-LINC02	si-LINC02	si-LINC02	si-LINC02587-iii
MMP2	20890.226	20598.357	24062.407	26215.117	24282.419	24388.709	19792.833	21823.085	21011.223	22785.409	20813.606	21064.97
N-Cadheri	20454.91	21485.14	23835.5	25964.4	24046.72	24637.62	18039.48	21309.48	19349.44	21241.86	18608.75	18725.53
ZEB1	19366.62	18606.35	19297.31	22219.67	21453.98	23144.13	13530.86	14375.86	11326.5	12697.33	13709.08	13266.83
ZO1	21543.2	22592.36	23835.5	25465.97	24989.53	26628.95	17037.57	15916.66	16753.78	18911.53	15913.93	16646.03
actin	21765.86	22144.47	22690.95	24971.54	23570.31	24891.54	25047.89	25675.1	23596.88	25902.51	24498.36	25993.79
MMP2/acti	0.95977	0.930181	1.060441	1.0498	1.030212	0.979799	0.7902	0.849971	0.890424	0.87966	0.849592	0.810385
N-Cadheri	0.9397703	0.9702258	1.0504407	1.0397597	1.0202121	0.9897991	0.7201996	0.8299669	0.82	0.8200695	0.7595918	0.7203847
ZEB1/acti	0.88977	0.840226	0.850441	0.8898	0.910212	0.929799	0.5402	0.559914	0.48	0.490197	0.559592	0.510385
ZO1/actin	0.98977	1.020226	1.050441	1.0198	1.060212	1.069799	0.6802	0.619926	0.71	0.730104	0.649592	0.640385
u87												
	Normal	Normal	Normal	si-NC	si-NC	si-NC	si-LINC02	si-LINC02	si-LINC02	si-LINC02	si-LINC02	si-LINC02587-iii
MMP2	24957.83	24629.45	24561.79	24936.72	25576.96	25112.2	11613.66	14403.75	12397.12	15886.07	16617.33	18375.26
N-Cadheri	16543.7	16252.23	17677.16	18175.09	16643.71	16265.54	15977.27	14929.5	13568.19	13635.2	14671.84	14034.3
ZEB1	14169.58	15946.98	16150.53	15279.87	15532.68	16019.25	13398.68	13918	13568.19	14896.8	12264.98	13298.25
ZO1	25182.58	26976.06	24769.12	25441.13	25322.4	27328.87	19349.44	19966.95	20019.96	20680.13	20688.99	20182.72
actin	22475.52	23466.14	22733.14	24220.12	23455.93	24629.61	25785.92	25287.28	23821.38	25231.86	24068.59	25535.09
MMP2/acti	1.110445	1.049574	1.08044	1.029587	1.090426	1.019594	0.450388	0.569605	0.52042	0.629604	0.690415	0.719608
N-Cadheri	0.736077	0.692582	0.777594	0.750413	0.709574	0.660406	0.619612	0.590395	0.56958	0.540396	0.609585	0.549608
ZEB1/acti	0.630445	0.679574	0.71044	0.630875	0.662207	0.650406	0.519612	0.550395	0.56958	0.590396	0.509585	0.520783
ZO1/actin	1.120445	1.149574	1.08956	1.050413	1.079574	1.109594	0.750388	0.789605	0.84042	0.819604	0.859585	0.790392

LN229

replicate blots 1 replicate blots 2 replicate blots 3

72kDa

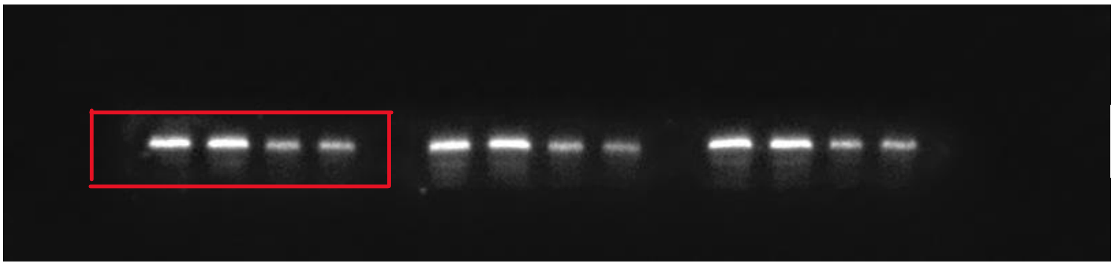

MMP2

replicate blots 1 replicate blots 2 replicate blots 3

72kDa

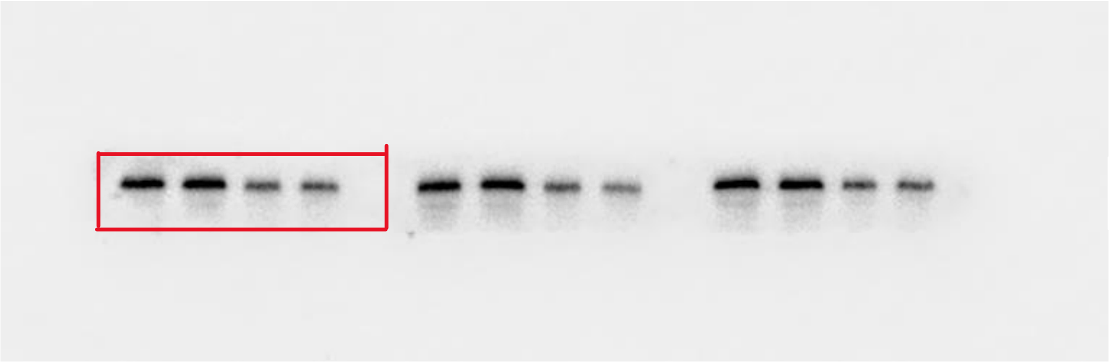

MMP2

replicate blots 1 replicate blots 2 replicate blots 3

140kDa

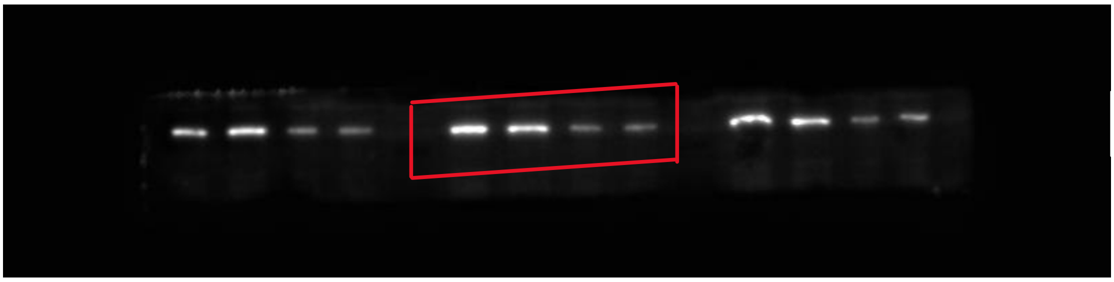

N-cadherin

replicate blots 1 replicate blots 2 replicate blots 3

140kDa

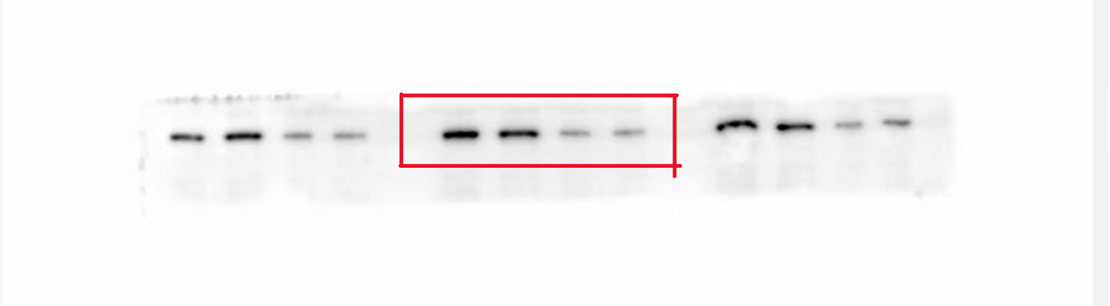

N-cadherin

replicate blots 1 replicate blots 2 replicate blots 3

200kDa

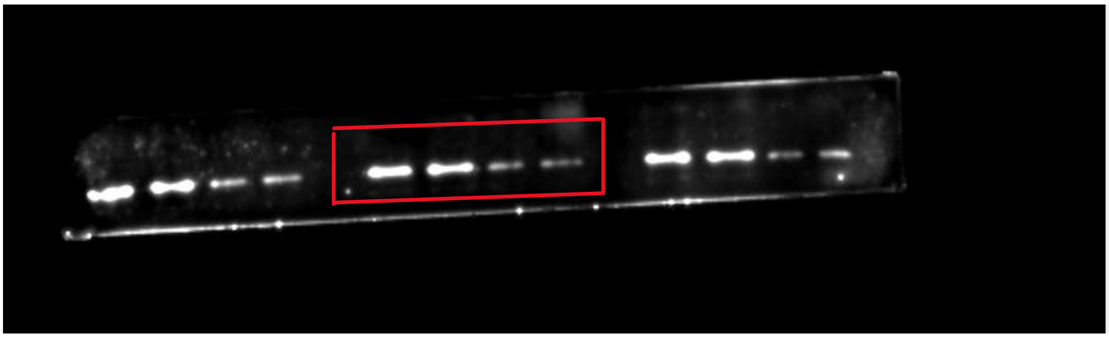

ZEB1

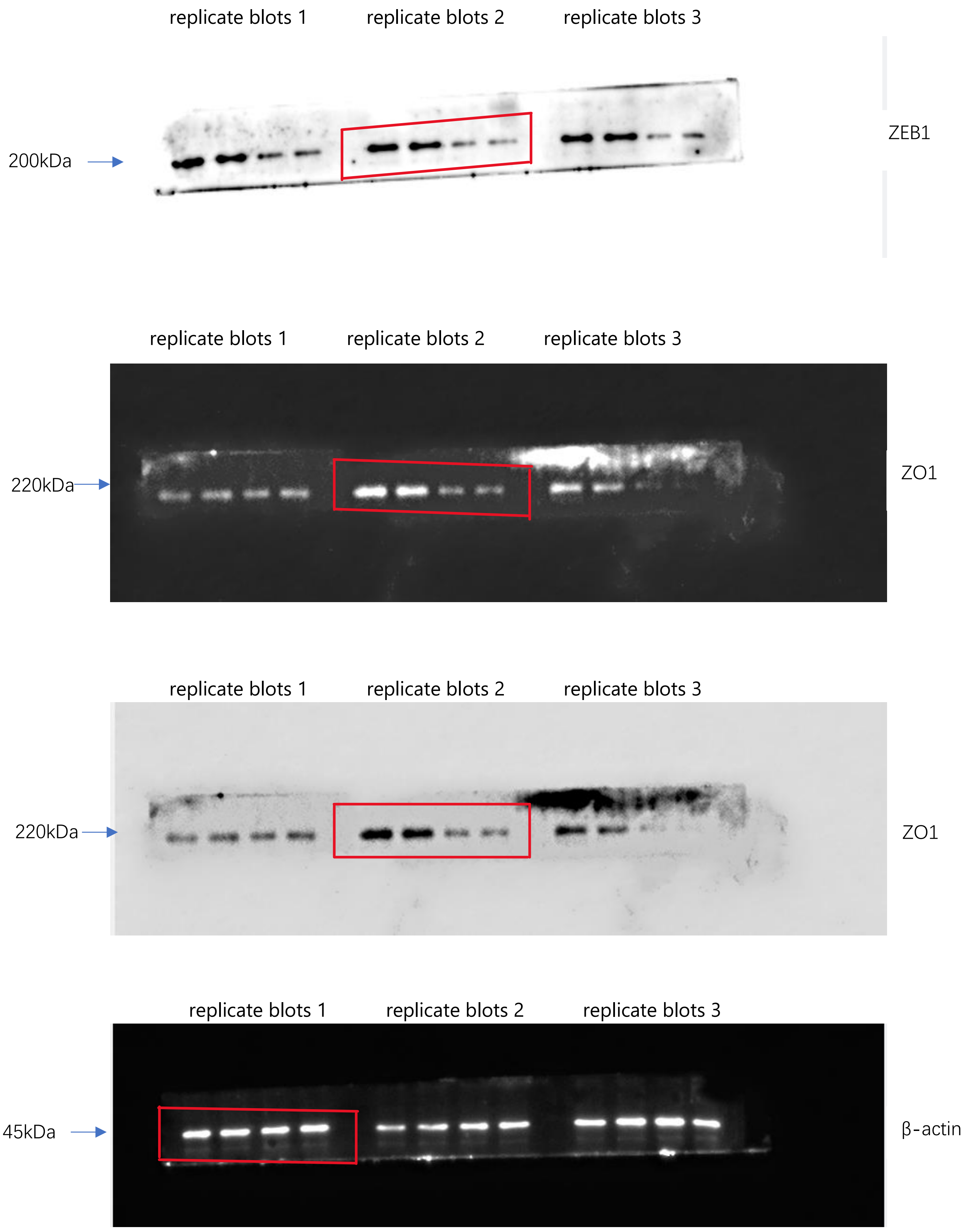

replicate blots 1
replicate blots 2
replicate blots 3
200kDa
ZEB1
replicate blots 1
replicate blots 2
replicate blots 3
220kDa
ZO1
replicate blots 1
replicate blots 2
replicate blots 3
220kDa
ZO1
replicate blots 1
replicate blots 2
replicate blots 3
45kDa
β-actin

replicate blots 1 replicate blots 2 replicate blots 3

45kDa → 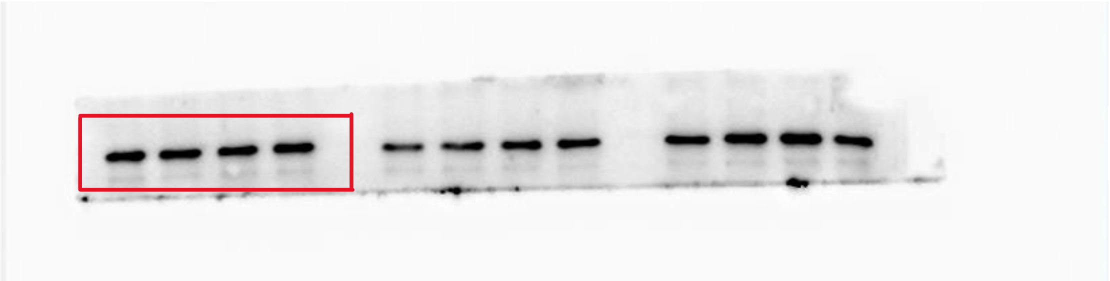 β-actin

U87

replicate blots 1 replicate blots 2 replicate blots 3

72kDa → 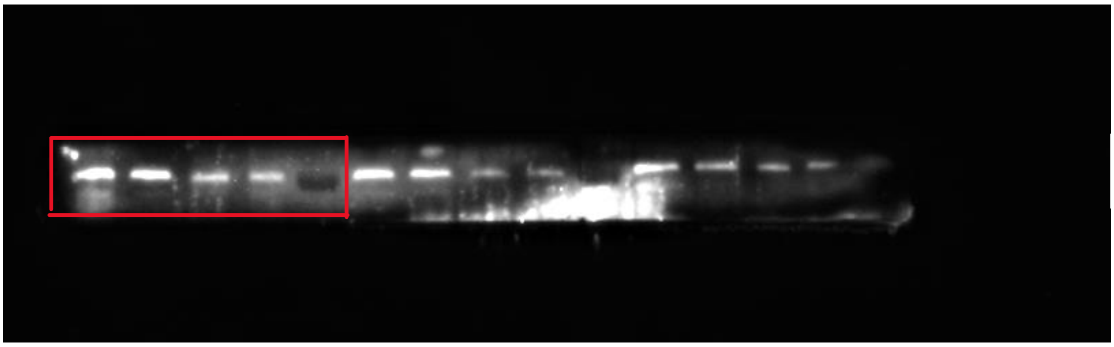 MMP2

replicate blots 1 replicate blots 2 replicate blots 3

72kDa → 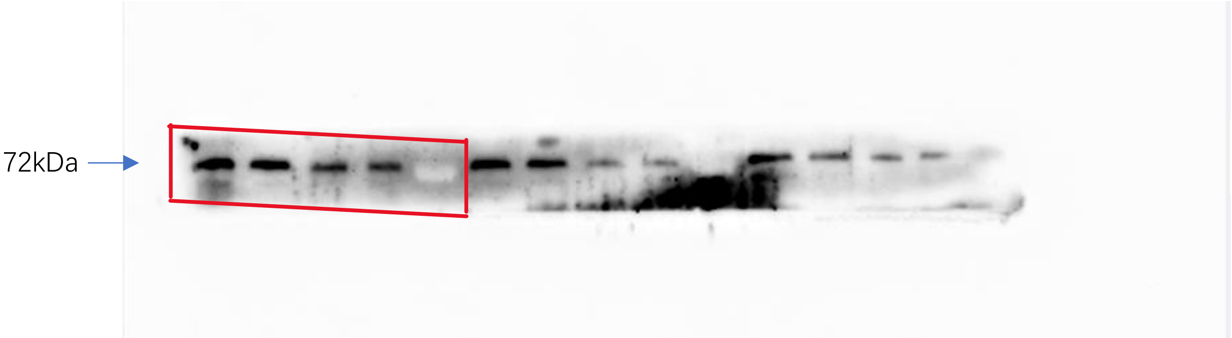 MMP2

replicate blots 1 replicate blots 2 replicate blots 3

140kDa → 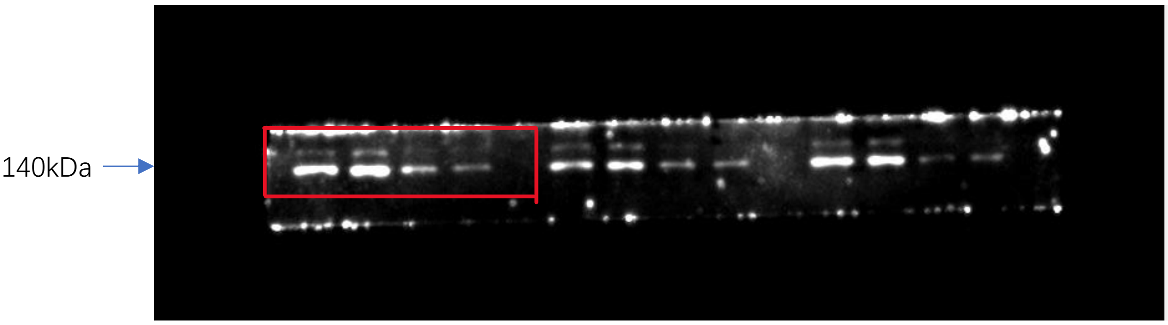 N-cadherin

replicate blots 1 replicate blots 2 replicate blots 3

140kDa

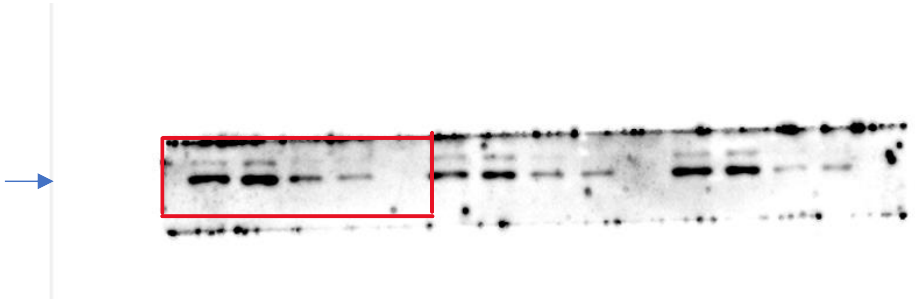

N-cadherin

replicate blots 1 replicate blots 2 replicate blots 3

200kDa

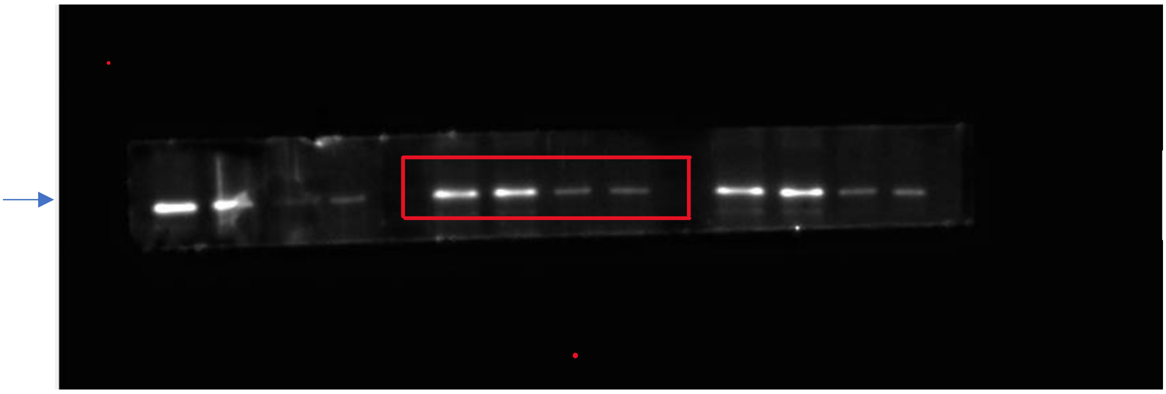

ZEB1

replicate blots 1 replicate blots 2 replicate blots 3

200kDa

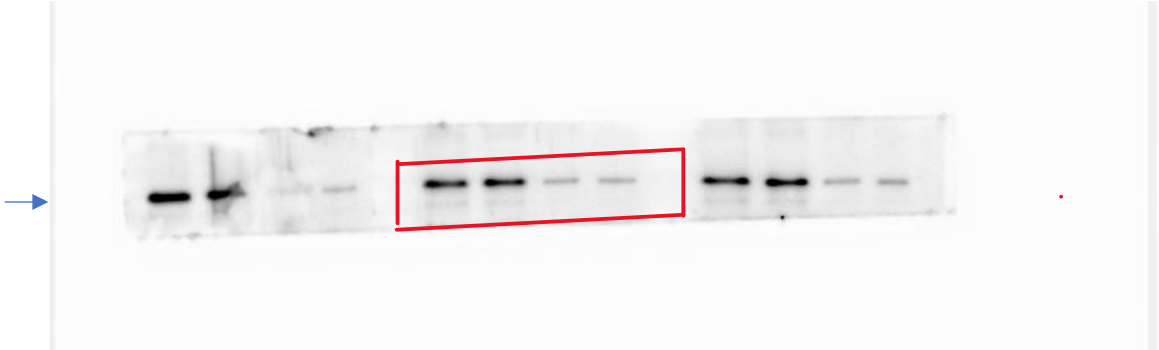

ZEB1

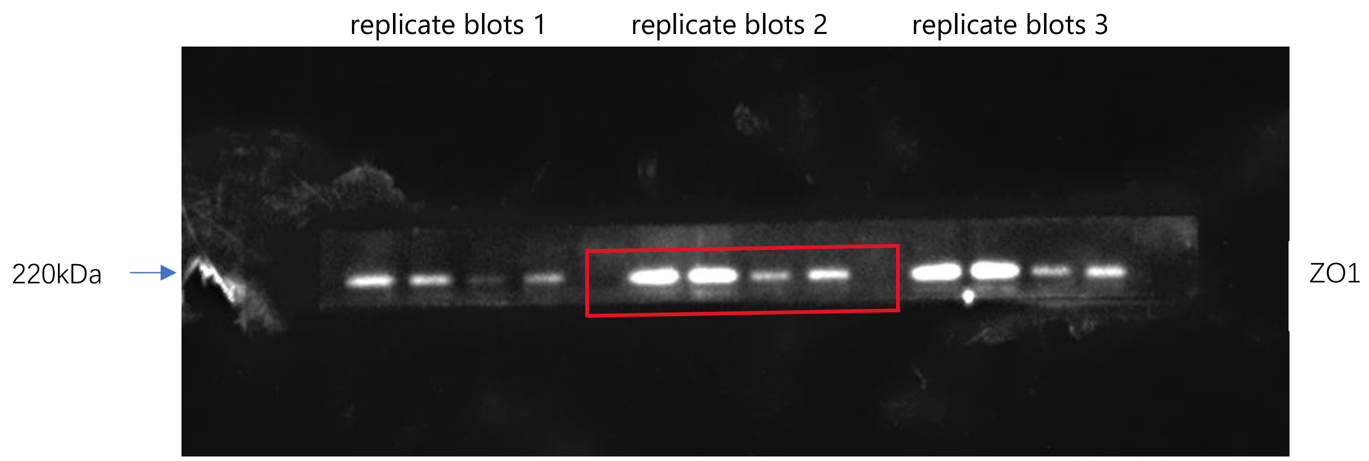
replicate blots 1
replicate blots 2
replicate blots 3
220kDa
ZO1

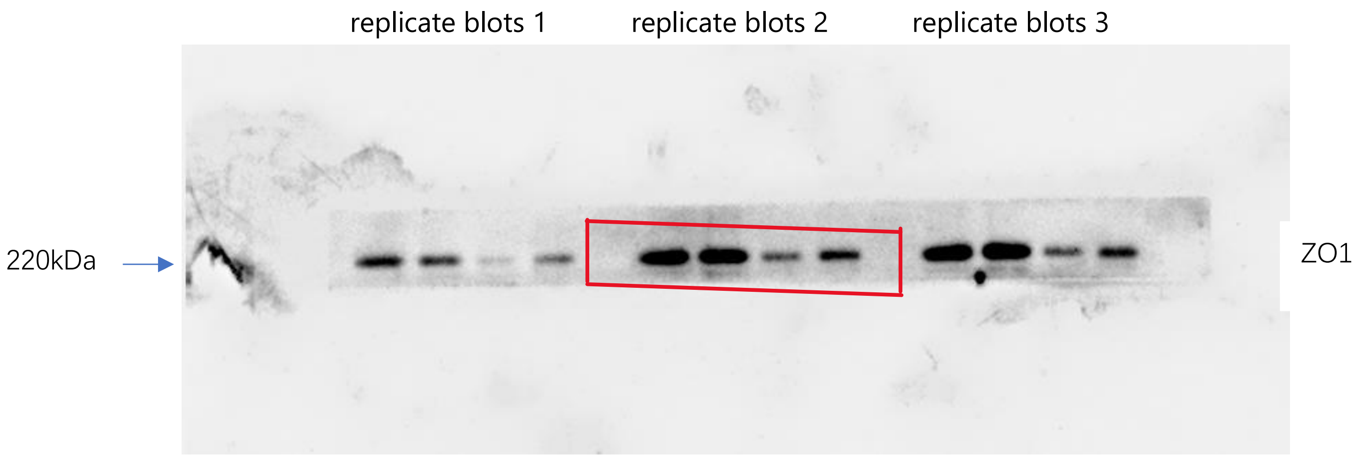
replicate blots 1
replicate blots 2
replicate blots 3
220kDa
ZO1

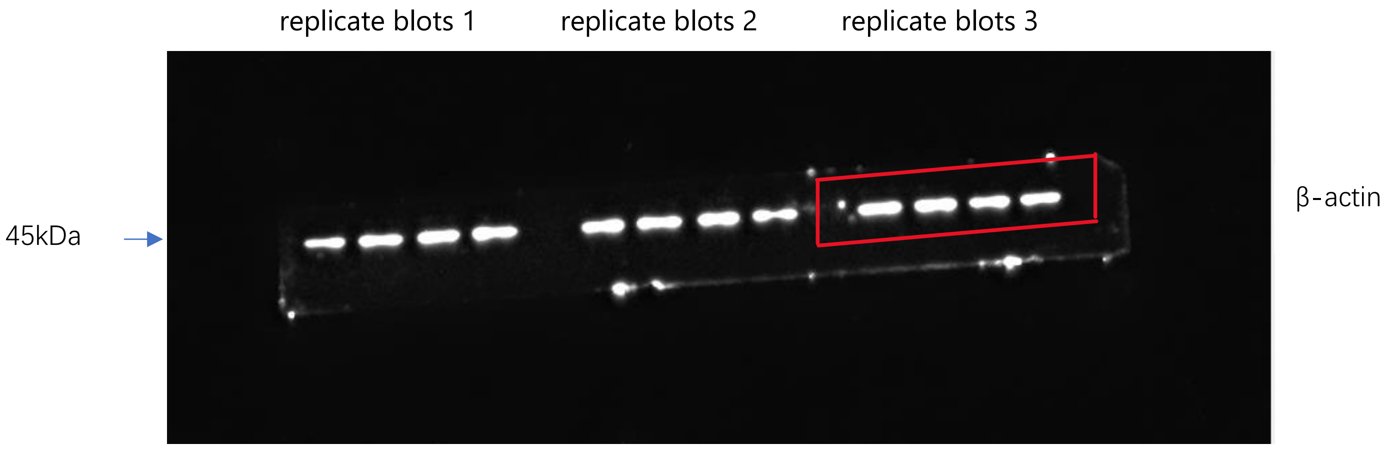
replicate blots 1
replicate blots 2
replicate blots 3
45kDa
β-actin

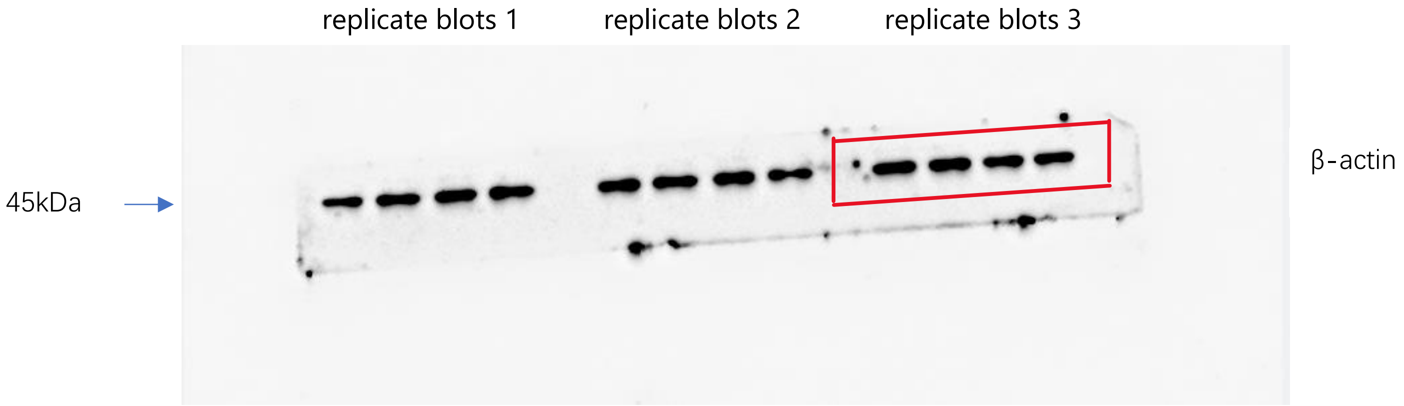
replicate blots 1
replicate blots 2
replicate blots 3
45kDa
β-actin

Supplementary figures 5

Genes associated with ferroptosis+PCR

LN229	NC	NC	NC	si-LINC02	si-LINC02	si-LINC02587-ii
FTH1	31.63	32.73	32.26	31.33	32.17	31.81
STEAP3	35.09	35.86	34.58	29.57	29.12	29.08
ACSL5	35.45	34.56	36.89	33.93	33.98	34.58
ATG5	33.86	34.57	34.92	29.23	29.51	29.63
SLC39A14	34.5	35.18	34.89	33.84	32.99	34.31
HMOX1	33.46	34.85	33.02	29.23	29.56	29.14
TF	26.76	27.88	27.87	31.14	30.01	30.94
SLC3A2	27.39	26.88	27.69	29.54	30.76	30.13
AIFM2	27.42	27.81	28.47	32.79	31.78	33.21
ACSL3	27.1	28.26	27.46	30.78	29.67	30.43
ACSL1	26.55	26.64	25.82	28.96	28.85	29.2
PRNP	28.67	29.29	27.78	30.99	31.87	31.45
GAPDH	14.98	15.03	15.36	16.27	16.12	16.20

U87	NC	NC	NC	si-LINC02	si-LINC02	si-LINC02587-ii
FTH1	34.25	33.43	35.67	32.02	31.97	31.78
STEAP3	33.18	33.75	32.68	26.85	26.97	27.36
ACSL5	30.54	31.62	31.25	30.29	31.15	30.86
ATG5	33.42	34.32	33.75	32.74	32.59	33.29
SLC39A14	32.78	33.46	33.92	28.85	28.64	29.54
HMOX1	32.43	33.76	32.14	27.97	28.41	28.87
TF	25.57	26.35	26.69	31.36	29.51	29.73
SLC3A2	27.32	28.13	27.32	29.98	30.76	30.34
AIFM2	26.26	26.67	27.43	31.68	30.66	32.19
ACSL3	27.76	28.76	26.96	30.32	30.72	30.58
ACSL1	25.37	25.58	25.89	27.96	28.23	28.02
PRNP	26.48	25.69	26.54	28.49	29.32	28.51
GAPDH	15.16	15.05	15.69	16.35	16.57	16.49

GSH level of cells

LN229

LN229						
NC+DMSO		absorbance		mean	ug/ml	ug/10^6 cell
	0.1463	0.1505	0.1499	0.1489	42.63813	5.542956
	0.1559	0.1528	0.1476	0.1521	43.73013	5.684916
	0.1587	0.1613	0.1625	0.160833	46.71038	6.072349
NC+iFSP1		absorbance		mean	ug/ml	ug/10^6 cell
	0.1338	0.1345	0.1402	0.136167	38.29288	4.978074
	0.1417	0.1406	0.1433	0.141867	40.238	5.23094
	0.1417	0.1462	0.1431	0.143667	40.85225	5.310793
		absorbance		mean	ug/ml	ug/10^6 cell
si-ii+DMSO	0.1421	0.1459	0.1418	0.143267	40.71575	5.293048
	0.1341	0.1337	0.1415	0.136433	38.38388	4.989904
	0.1416	0.1465	0.1437	0.143933	40.94325	5.322623
		absorbance		mean	ug/ml	ug/10^6 cell
si-ii+iFSP1	0.1388	0.1402	0.1423	0.140433	39.74888	5.167354
	0.1335	0.1332	0.1408	0.135833	38.17913	4.963286
	0.1427	0.1416	0.1402	0.1415	40.11288	5.214674

U87

u87						
NC+DMSO		absorbance		mean	ug/ml	ug/10^6 cell
	0.1981	0.2044	0.2126	0.205033	59.72321	7.764017
	0.1979	0.1956	0.2182	0.2039	59.43565	7.726634
	0.1854	0.1932	0.2011	0.193233	56.72919	7.374795
NC+iFSP1		absorbance		mean	ug/ml	ug/10^6 cell
	0.1796	0.1853	0.1785	0.181133	53.65906	6.975678
	0.1874	0.1867	0.1893	0.1878	55.35059	7.195577
	0.1923	0.1915	0.1867	0.190167	55.95109	7.273641
si-ii+DMSO		absorbance		mean	ug/ml	ug/10^6 cell
	0.1912	0.1923	0.1841	0.1892	55.70582	7.241756
	0.1869	0.1871	0.1897	0.1879	55.37597	7.198876
	0.1805	0.1861	0.1758	0.1808	53.57448	6.964683
si-ii+iFSP1		absorbance		mean	ug/ml	ug/10^6 cell
	0.1908	0.1919	0.1838	0.188833	55.61278	7.229662
	0.1862	0.1869	0.1891	0.1874	55.2491	7.182383
	0.1816	0.1848	0.1749	0.180433	53.48145	6.952588

229												
	Normal	Normal	Normal	si-NC	si-NC	si-NC	-LINC02587	-LINC02587	-LINC02587	-LINC02587	-LINC02587	-LINC02587-iii
FSP1	18216. 43	18421. 21	18946. 71	19266. 41	20634. 28	20857. 61	18559. 65	18141. 33	16910. 51	14833. 02	12594. 24	16794. 55
CoQ10B	23241. 59	24746. 62	21822. 91	25911. 71	25677. 99	28511. 61	21247. 36	20651. 99	16659	17101. 21	14268. 28	17073. 09
actin	21935. 46	24251. 59	22278. 48	23126. 53	23566. 96	24570. 35	24433. 75	25482. 7	23151. 39	25157. 66	24200. 47	25853. 16
FSP1/acti	0.8304559	0.7595877	0.8504489	0.8330868	0.8755597	0.8488935	0.7595907	0.7119078	0.7304319	0.5896025	0.5204132	0.6496132
CoQ10B/ac	1. 059544	1. 020412	0. 979551	1. 120432	1. 089576	1. 160407	0. 869591	0. 810432	0. 719568	0. 679762	0. 589587	0. 660387

u87												
	Normal	Normal	Normal	si-NC	si-NC	si-NC	-LINC02587	-LINC02587	-LINC02587	-LINC02587	-LINC02587	-LINC02587-iii
FSP1	26863. 21	23697. 14	25022. 04	24202. 9	25781. 17	25978. 14	10487. 98	12876. 17	10837. 2	11608. 01	12912. 39	10303. 91
CoQ10B	22568. 23	22136. 67	23645. 23	21852. 13	24097. 98	25476. 24	11257. 84	12101. 22	11970. 13	11112. 65	12424. 54	11810. 97
actin	23766. 56	22578. 23	22946. 83	23507. 67	24331. 29	24044. 57	24995. 19	25231. 7	23038. 72	25817. 79	24342. 24	25150. 99
FSP1/acti	1.1302945	1.0495571	1.0904358	1.0295746	1.059589	1.0804159	0.4195999	0.5103171	0.4703906	0.4496127	0.5304519	0.4096819
CoQ10B/ac	0. 949579	0. 980443	1. 030436	0. 929575	0. 990411	1. 059543	0. 4504	0. 479604	0. 519566	0. 430426	0. 510411	0. 469602

LN229

replicate blots 1 replicate blots 2 replicate blots 3

23kDa

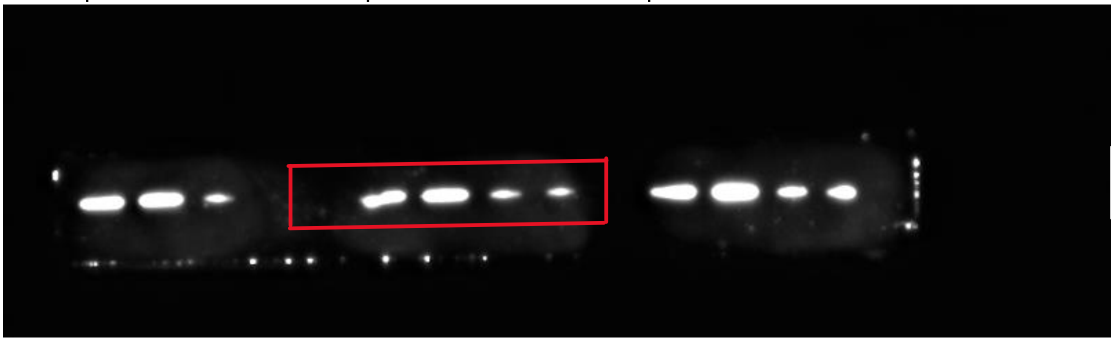

CoQ10B

replicate blots 1 replicate blots 2 replicate blots 3

23kDa

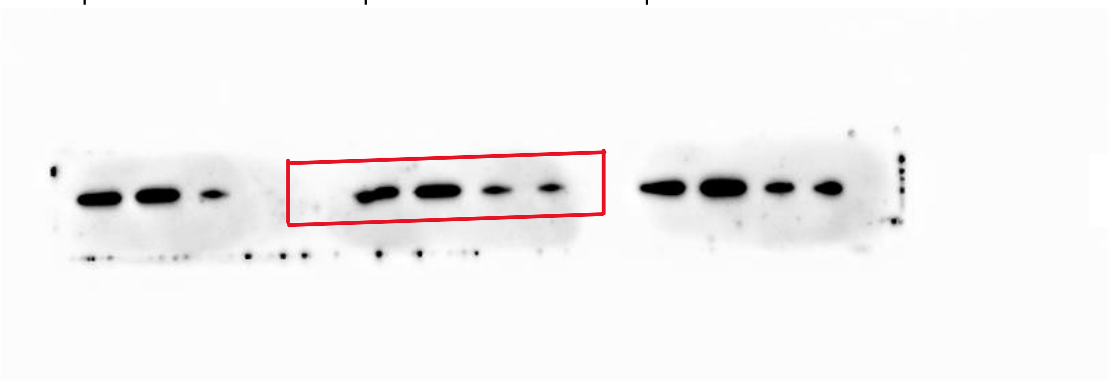

CoQ10B

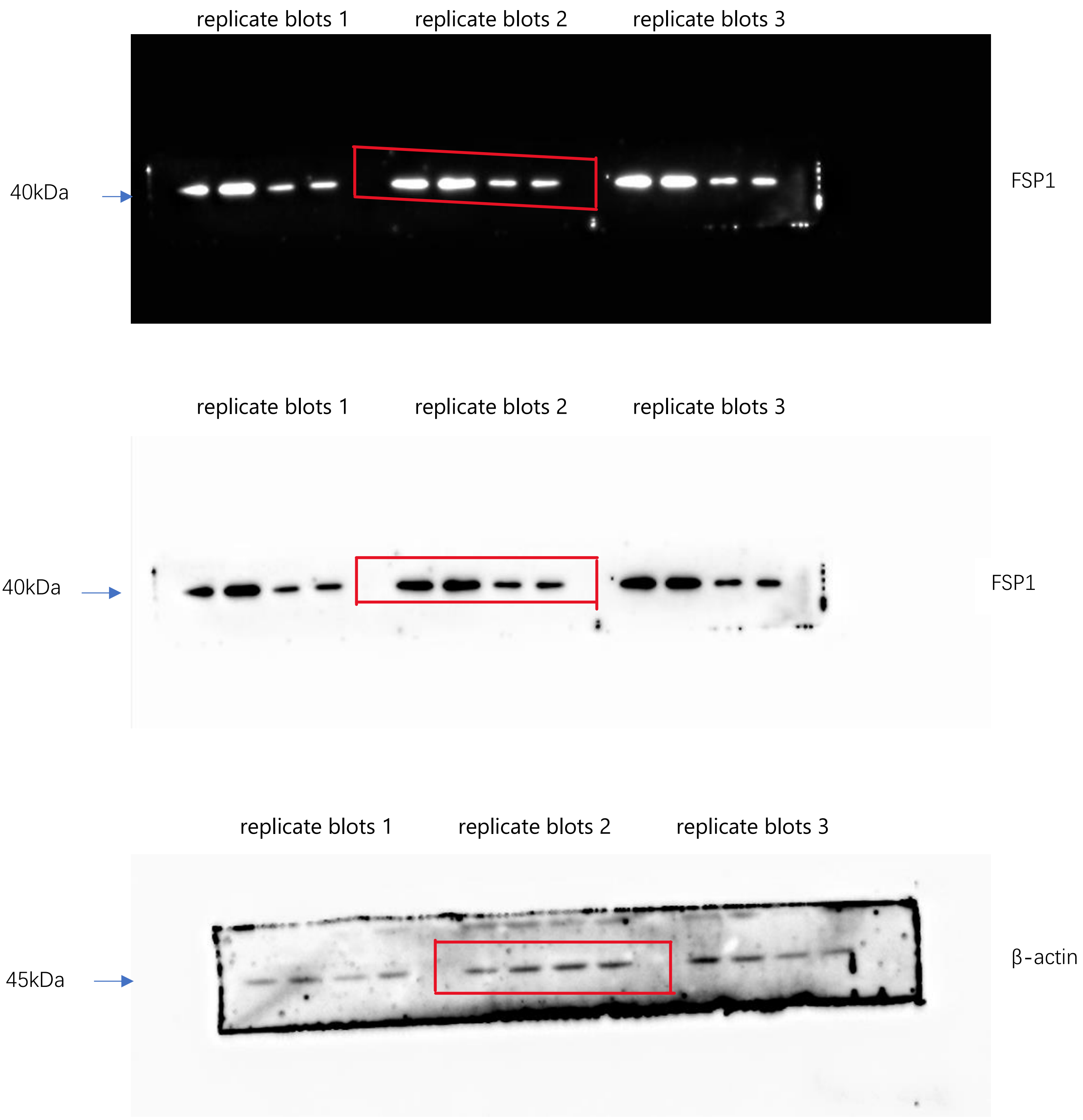
replicate blots 1
replicate blots 2
replicate blots 3
40kDa
FSP1
replicate blots 1
replicate blots 2
replicate blots 3
40kDa
FSP1
replicate blots 1
replicate blots 2
replicate blots 3
45kDa
β-actin

U87

replicate blots 1 replicate blots 2 replicate blots 3

23kDa →

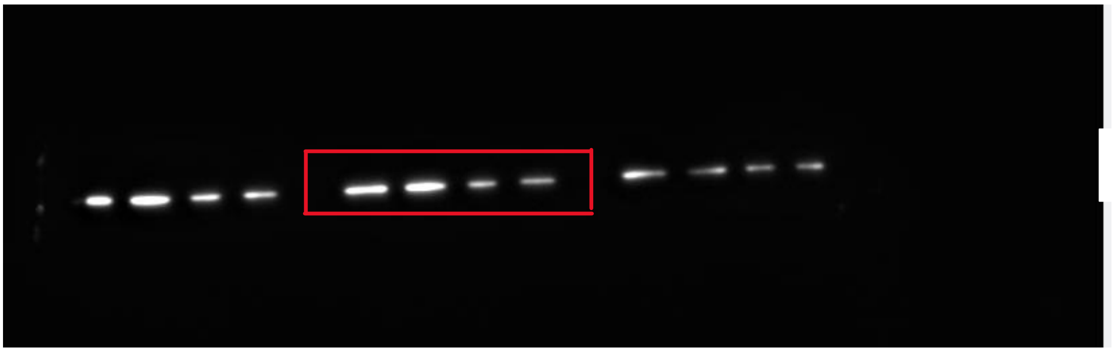

CoQ10B

replicate blots 1 replicate blots 2 replicate blots 3

23kDa →

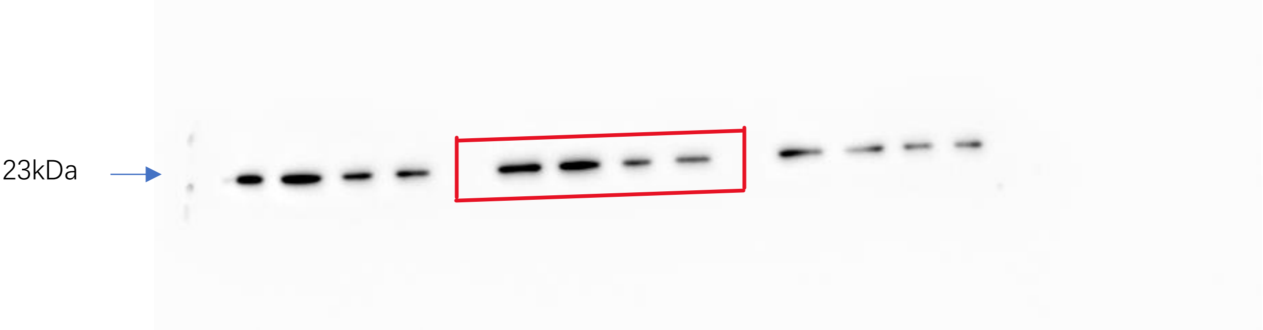

CoQ10B

replicate blots 1 replicate blots 2 replicate blots 3

40kDa →

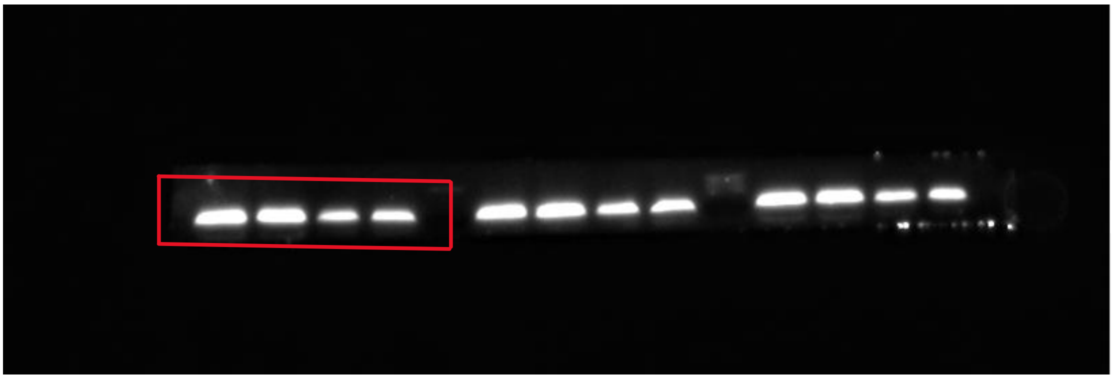

FSP1

replicate blots 1 replicate blots 2 replicate blots 3

40kDa

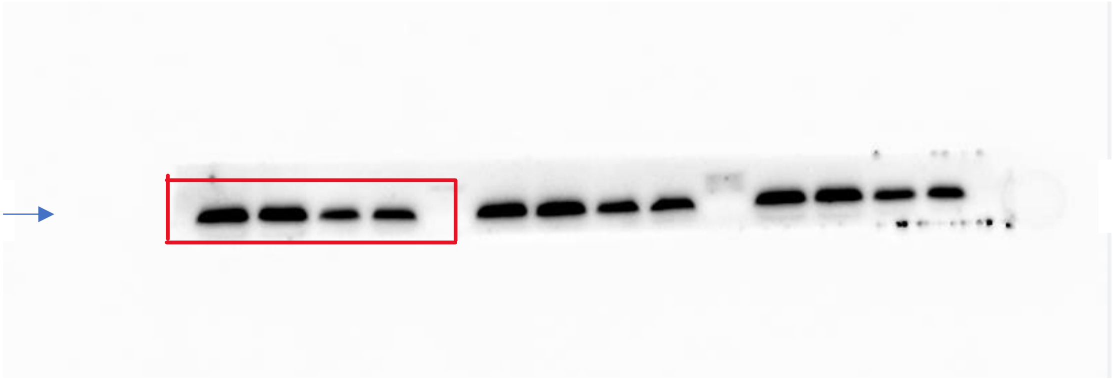

FSP1

replicate blots 1 replicate blots 2 replicate blots 3

45kDa

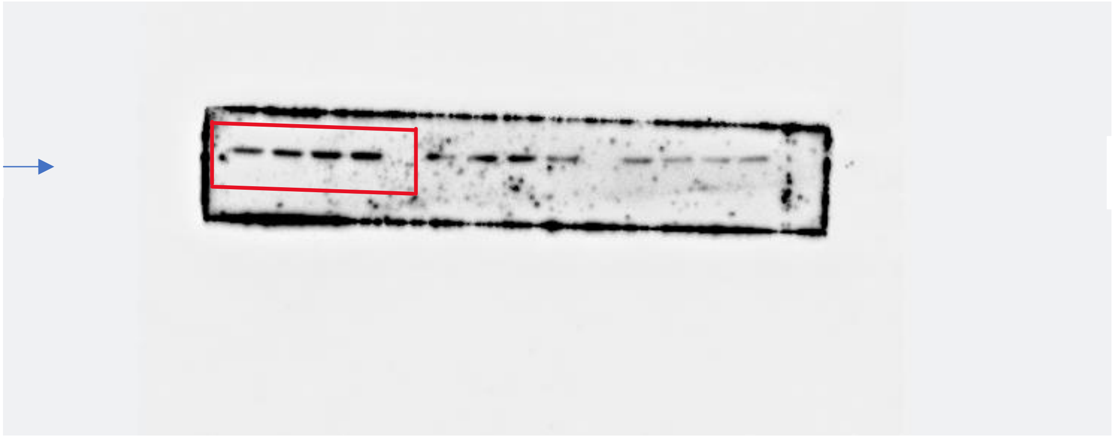

β-actin

Supplementary figures 6

flow cytometry - c11

LN229		
	LINC02587	
NC	si-1	si-2
11.3	14.3	20.2
11.8	15.3	21.2
13	15.8	21.7
U87		
	LINC02587	
NC	si-1	si-2
1.52	12.7	25
1.58	12.5	28
1.67	20	35.3

flow cytometry+Fe2+

229	nc	si-1	si-2		U87	nc	si-1	si-2
	158	293	246			162	570	584
	172	266	233			150	578	574
	158	280	218			135	560	571
MEAN	162.6666667					149		
	0.971311475	1.801229508	1.512295082			1.087248322	3.825503356	3.919463087
	1.057377049	1.635245902	1.432377049			1.006711409	3.879194631	3.852348993
	0.971311475	1.721311475	1.340163934			0.906040268	3.758389262	3.832214765

flow cytometry+JC-1

JC-1							
			LINC02587				
	229					U87	
NC	si-1	si-2			NC	si-1	si-2
38.8 %	47.6 %	50.3 %			13.4 %	19.6 %	33.3 %
39.9 %	48.3 %	50.4 %			13.8 %	22.9 %	36.4 %
40.5 %	49.3 %	50.4 %			13.9 %	26.4 %	37.3 %
NC	si-1	si-2			NC	si-1	si-2
38.8	47.6	50.3			13.4	19.6	33.3
39.9	48.3	50.4			13.8	22.9	36.4
40.5	49.3	50.4			13.9	26.4	37.3

GSH level of cells

LN229						
NC	absorbance			mean	ug/ml	ug/10^6 cell
	0.1557	0.1646	0.1701	0.163467	47.609	6.18917
	0.1458	0.1526	0.1465	0.1483	42.43338	5.516339
	0.1564	0.1513	0.1486	0.1521	43.73013	5.684916
si-linc02587-ii	absorbance			mean	ug/ml	ug/10^6 cell
	0.1413	0.1468	0.1413	0.143133	40.67025	5.287133
	0.1338	0.1342	0.1408	0.136267	38.327	4.98251
	0.1501	0.1513	0.1486	0.15	43.0135	5.591755
si-linc02587-iii	absorbance			mean	ug/ml	ug/10^6 cell
	0.1356	0.1223	0.1287	0.128867	35.80175	4.654228
	0.1139	0.1219	0.1215	0.1191	32.46888	4.220954
	0.1226	0.1124	0.1149	0.116633	31.62713	4.111526

u87						
NC	absorbance			mean	ug/ml	ug/10^6 cell
	0.2168	0.2266	0.2302	0.224533	64.67094	8.407223
	0.2209	0.2013	0.1997	0.2073	60.29833	7.838783
	0.2015	0.2095	0.1989	0.2033	59.28341	7.706843
si-linc02587-ii	absorbance			mean	ug/ml	ug/10^6 cell
	0.1905	0.1928	0.1828	0.1887	55.57895	7.225264
	0.1873	0.1872	0.1907	0.1884	55.50283	7.215368
	0.1802	0.1711	0.1743	0.1752	52.1536	6.779967
si-linc02587-iii	absorbance			mean	ug/ml	ug/10^6 cell
	0.1576	0.1747	0.1808	0.171033	51.09639	6.64253
	0.1645	0.1505	0.1603	0.158433	47.89939	6.226921
	0.1513	0.1524	0.1516	0.151767	46.20786	6.007021

Supplementary figures 7

tumor image

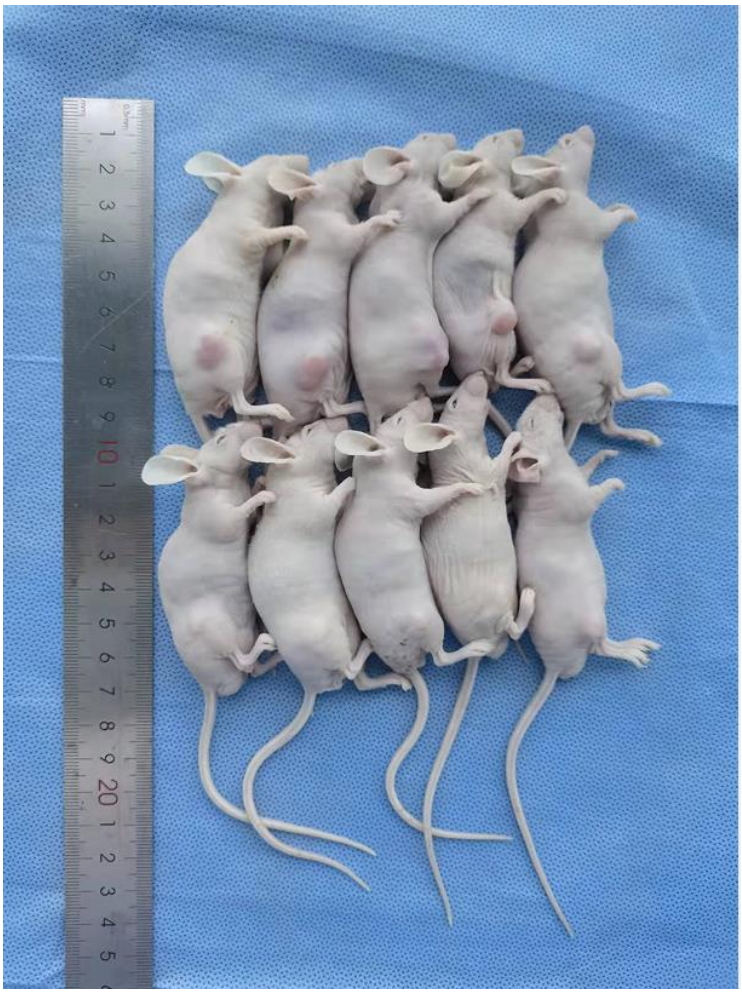

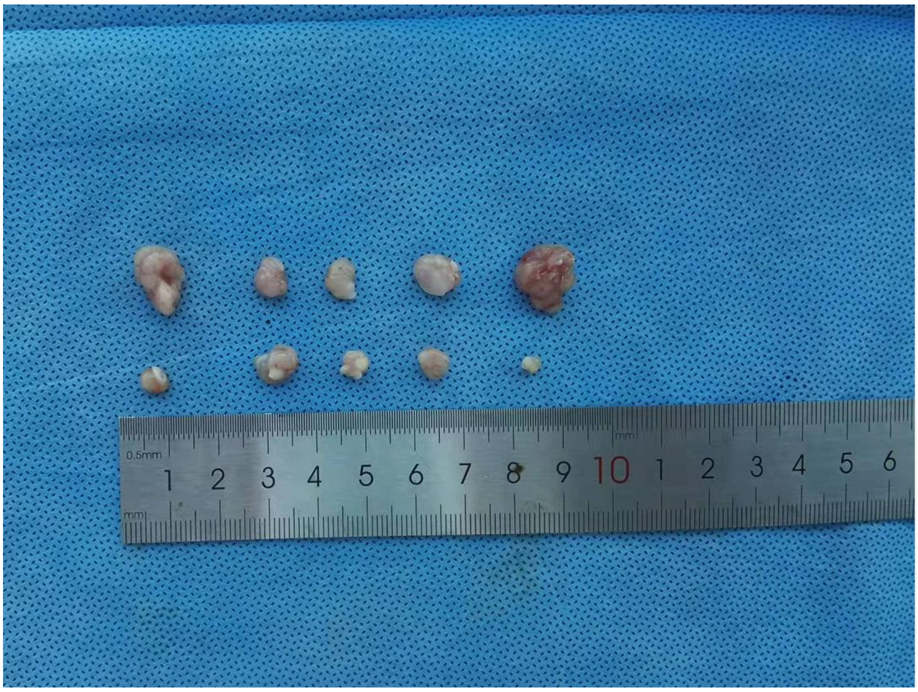

tumor volume

7D	LINC02587						
	NC	L	3.32	3.14	3.16	3.33	3.26
		W	3.3	3.13	3.15	3.32	3.22
		V	18.0774	15.381133	15.67755	18.352296	16.900492
	si-LINC02	L	3.2	3.16	3.29	3.38	3
		W	3.19	3.12	3.25	3.3	3
		V	16.28176	15.380352	17.3753125	18.4041	13.5
14D	LINC02587						
	NC	L	5.43	4.38	4.16	4.69	5.35
		W	5.39	4.35	4.15	4.66	5.27
		V	78.8764515	41.440275	35.8228	50.923082	74.2925075
	si-LINC02	L	3.26	3.58	3.36	3.72	3.92
		W	3.24	3.56	3.36	3.69	3.86
		V	17.111088	22.685744	18.966528	25.325946	29.203216
21D	LINC02587						
	NC	L	7.25	5.37	5.56	6.35	7.89
		W	7.36	5.35	5.48	6.34	7.87
		V	196.3648	76.8514125	83.484512	127.62103	244.3410705
	si-LINC02	L	4.98	5.58	5.02	5.68	4.12
		W	4.96	5.57	5	5.56	4.12
		V	61.257984	86.559471	62.75	87.794624	34.967264
28D	LINC02587						
	NC	L	10.44	6.38	6.42	7.1	10.13
		W	10.23	6.31	6.28	7.1	10.1
		V	546.288138	127.013359	126.597264	178.9555	516.68065
	si-LINC02	L	5.55	6.64	5.67	6.08	4.44
		W	5.48	6.53	5.56	5.98	4.41
		V	83.33436	141.567788	87.640056	108.711616	43.174782
35D	LINC02587						
	NC	L	11.63	8.45	8.62	9.14	12.38
		W	11.2	8.3	8.4	9.1	12.1
		V	729.4336	291.06025	304.1136	378.4417	906.2779
	si-LINC02	L	6.73	7.84	6.78	7.09	5.54
		W	6.6	7.5	6.3	6.9	5.4
		V	146.5794	220.5	134.5491	168.77745	80.7732

tumor weight

NC(g)	0.73	0.54	0.52	0.65	0.76
si-LINC02[illegible]	0.38	0.56	0.36	0.41	0.32

Ki67(%)			
	NC	sh-LINC02587	
	60	40	
	55	44	
	63	37	
FSP1(%)			
	NC	sh-LINC02587	
	85	64	
	89	70	
	90	73	

IHC-image

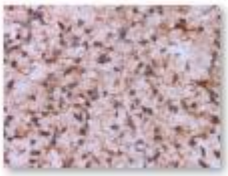
NC-FSP1-1

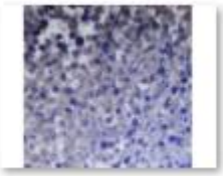
NC-FSP1-2

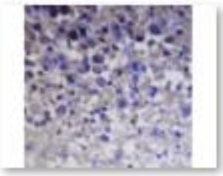
NC-FSP1-3

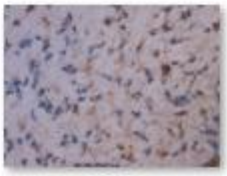
shRNA-FSP1-1

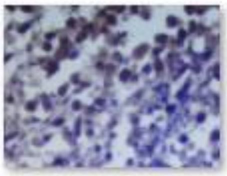
shRNA-FSP1-2

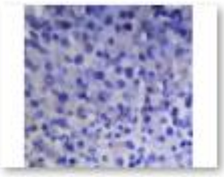
shRNA-FSP-3

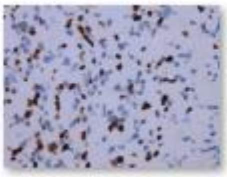
NC-Ki67-1

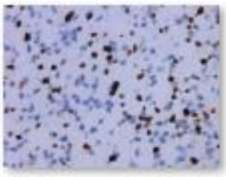
NC-ki67-2

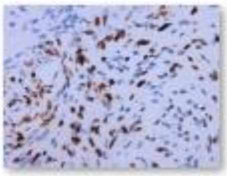
NC-ki67-3

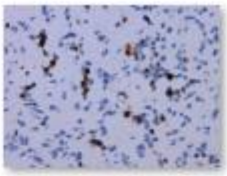
shRNA-Ki67-1

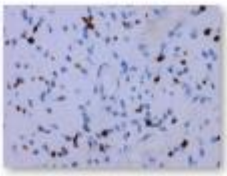
shRNA-Ki67-2

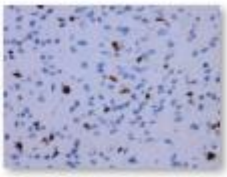
shRNA-Ki67-3
